# The genomic underpinnings of apoptosis in the silkworm, *Bombyx mori*

**DOI:** 10.1186/1471-2164-11-611

**Published:** 2010-10-31

**Authors:** Jin-Ye Zhang, Min-Hui Pan, Zhi-Ya Sun, Shu-Jing Huang, Zi-Shu Yu, Di Liu, Dan-Hong Zhao, Cheng Lu

**Affiliations:** 1 The Key Sericultural Laboratory of Agricultural Ministry, Institute of Sericulture and Systems Biology, Southwest University, Chongqing 400715, China

## Abstract

**Background:**

Apoptosis is regulated in an orderly fashion by a series of genes, and has a crucial role in important physiological processes such as growth development, immunological response and so on. Recently, substantial studies have been undertaken on apoptosis in model animals including humans, fruit flies, and the nematode. However, the lack of genomic data for silkworms limits their usefulness in apoptosis studies, despite the advantages of silkworm as a representative of Lepidoptera and an effective model system. Herein we have identified apoptosis-related genes in the silkworm *Bombyx mori *and compared them to those from insects, mammals, and nematodes.

**Results:**

From the newly assembled genome databases, a genome-wide analysis of apoptosis-related genes in *Bombyx mori *was performed using both nucleotide and protein Blast searches. Fifty-two apoptosis-related candidate genes were identified, including five caspase family members, two tumor necrosis factor (TNF) superfamily members, one Bcl-2 family member, four baculovirus IAP (inhibitor of apoptosis) repeat (BIR) domain family members and 1 RHG (Reaper, Hid, Grim, and Sickle; *Drosophila *cell death activators) family member. Moreover, we identified a new caspase family member, *BmCaspase-New*, two splice variants of *BmDronc*, and Bm3585, a mammalian TNF superfamily member homolog. Twenty-three of these apoptosis-related genes were cloned and sequenced using cDNA templates isolated from BmE-SWU1 cells. Sequence analyses revealed that these genes could have key roles in apoptosis.

**Conclusions:**

*Bombyx mori *possesses potential apoptosis-related genes. We hypothesized that the classic intrinsic and extrinsic apoptotic pathways potentially are active in *Bombyx mori*. These results lay the foundation for further apoptosis-related study in *Bombyx mori*.

## Background

Programmed cell death [1], as well as cell proliferation and cell differentiation, has a crucial role in biological growth and development. There are two primary programmed cell death signaling pathways [2], apoptosis [3] and autophagy [4], of which apoptosis has been researched more extensively. Apoptosis is characterized by morphological changes and biochemical events such as cytoplasmic and nuclear condensation, phosphatidylserine extrusion, vacuolization, chromatin condensation, DNA fragmentation, and formation of apoptotic bodies that are ultimately engulfed by surrounding cells or macrophages [2]. Cell death by autophagy involves cell degradation by internal lysozymes [4]. There are very important connections between apoptotic cell death and autophagic cell death, as they occur concurrently in many processes [5].

Apoptotic mechanisms are being clarified in model organisms using completed genome sequences, especially in nematodes, fruit flies and humans. Compared to *Drosophila *and humans, the apoptosis network in nematodes is much simpler [6]. There are several differences in apoptotic mechanisms between mammals and nematodes. In mammals, two primary apoptotic signaling pathways have been described: the extrinsic pathway, which is initiated by the tumor necrosis factor (TNF)/nerve growth factor (NGF) receptors superfamily [7-11], and the intrinsic pathway, which include the mitochondrial pathway, the endoplasmic reticulum pathway and the DNA damage pathway [12-15]. The intrinsic and extrinsic pathways are connected by caspase-mediated activation of the pro-apoptotic Bcl-2 family member BID and the c-Jun N-terminal kinases (JNK), which converge on effector caspase activation [16-19]. Most insect apoptosis research has used *Drosophila*. There are some fundamental differences in apoptotic signaling pathways between *Drosophila *and mammals. For example, the absence of RHG (Reaper, Hid, Grim and Sickle) family proteins virtually blocks apoptosis [20]. Although Smac/Dablo and Htra2/Omi are functional homologs of the RHG family, their apoptotic roles are not as critical in vertebrates as RHG is in *Drosophila *[21]. Furthermore, cytochrome-c is dispensable for caspase activation [22-26] and it is unclear whether mitochondria participate in apoptosis [27,28] in *Drosophila*. Kumarswamya and colleagues [29] recently used Sf9 cells to demonstrate that cyosolic cytochrome-c release is an essential event for caspase activation during Lepidopteran apoptosis, and that cytochrome-c release might occur independent of mitochondrial membrane potential loss and permeability transition pore formation. Furthermore, cytochrome-c has been detected by Western blot in the cytoplasm of UV-induced apoptotic silkworm cells, BmE-SWU1 [30]. These all are distinctively different from the mechanisms reported in *Drosophila*, but are similar to mammalian apoptosis.

The domestic silkworm *Bombyx mori *(a representative of Lepidoptera) has important economic value. Investigation into apoptosis in Lepidotera began at the same time as *Drosophila *[31,32]. Since intersegmental muscle apoptosis was studied in 1965 [31], apoptosis research in silkworm has lagged far behind that of other organisms until the 1990s. Now, apoptosis research in silkworms mainly focus on two aspects. First, the morphological changes in tissues and cells during apoptosis-induced by extrinsic factors (such as ecdysone [33] or hemolymph [34-41]) and intrinsic factors (such as actinomycin D, ultraviolet light, and viruses [42-46]), the individual organs (e.g. wing or palea) in *Bombyx mori *apoptotic mutants [47] and tissues (midgut, silk gland) during metamorphosis [48]. The second aspect is gene cloning and identification. Tambunan [49] found that BmP109 in *Bombyx mori *contains the four conserved Bcl-2 homology (BH) regions, BH1, BH2, BH3, and BH4. Huang and colleagues [50] have cloned the IAP (inhibitor of apoptosis) homolog *BmIAP *from *Bombyx mori *BmN cells, and found that BmIAP inhibits apoptosis induced by Bax, but not Fas, in mammalian cells. Their biochemical data also suggested that BmIAP is a specific inhibitor of mammalian Caspase-9, but not the downstream effectors caspase-3 and caspase-7. In the same year, Kim and colleagues [34] reported that the 30 K protein from silkworm hemolymph inhibits virus- or chemical-induced apoptosis in human cells as well as insect cells, although the mechanism remains unknown. The first caspase family member identified was *BmCaspase-1 *in BmN cells [51]. Subsequently, the caspase family members *BmICE *[52], *BmICE-2 *and *BmICE-5 *[53] were cloned from *Bombyx mori *midgut and BmE cells. Recently, Bryant and colleagues [54] demonstrated both *Drosophila *DmReaper and its *Bombyx mori *ortholog *BmReaper *possessed conservative IAP binding and GH3 (Grim Helix 3) motifs. The *Bombyx mori *homologs *BmPkc *[55], *BmIcad *[56], and *BmCdc2 *[57] also have been cloned. However, how these genes participate in apoptosis in the silkworm is still unclear, and this area of research has been very fragmented.

Apoptotic mechanisms in model organisms (such as the nematode, *Drosophila *and mammals) can not accurately reflect the apoptotic mechanisms in silkworm. For these reasons, comprehensive and in-depth apoptosis research in *Bombyx mori *is urgent. Fortunately, the completion of the silkworm genome sequence [58] and a whole-genome chip [59] provide important tools for apoptosis research in *Bombyx mori*. Herein we looked for possible apoptosis-related homologs using information analysis in 9× genome sequencing data. Genes of interest were cloned and verified using cDNA templates isolated from the BmE cell line and different developmental stages of *Bombyx mori*. Finally, the potential apoptotic pathways in which these genes may act in *Bombyx mori *were analyzed.

## Results

### Identification of silkworm apoptosis-related genes

We have identified 52 apoptosis-related genes, including five members of the caspase family (*BmICE*, *BmICE-2 *and *BmICE-5 *are splice variants of *BmICE*; *BmDroncL *and *BmDroncS *are splice variants of *BmDronc*). We have also identified one member of the Bcl-2 family, two members of the TNF superfamily (TNFSF), and four members of the baculovirus IAP repeat (BIR) domain family in *Bombyx mori *(Table 1). Seventeen genes (*BmAkt, BmCaspase-1, BmICE, BmDredd, BmCdc2, BmCyt C, BmErk, BmICAD, BmIAP, BmICE-2, BmICE-5, BmJnk, BmPdk, BmPka, BmPkc, BmRas, BmReaper, BmSir2*, and *BmStat*) have been previously included in NCBI, among which ten genes have been reported in *Bombyx mori *including *BmCaspase-1 *[51], *BmICE *[52], *BmCdc2 *[57], *BmErk *[60], *BmICAD *[56], *BmIAP *[50], *BmJnk *[61], *BmPkc *[55], *BmReaper *[56], *BmCyt C *[30], *BmICE-2 *and *BmICE-5 *[53] (Additional file 1 Table A). Thirty five apoptosis genes were identified (Table 1) and accepted by the NCBI (Gene IDs are provided in detail in Additional file 1 Table B). These silkworm apoptosis-related genes are located on most of the 28 chromosomes, except the chromosomes 1, 6, 19, 24, 27, and 28. The number of exons in the genes varied from one to dozens. The comparing key apoptosis-related gene numbers in various species (Table 2) shows that there are fewer homologous genes in insects than in the higher eukaryotes. Many important genes in apoptosis pathways were cloned and identified, such as *BmApaf-1*, *BmP53*, *BmHtra2*, and *BmEndo G *(Table 1 and Additional file 2). However, we did not find homolog hits for many genes in our silkworm databases, including almost all genes of the Bcl-2 and TNFSF families, and *caspase-6*/*-7*, *Hid*, *Grim*, and *Sickle *of the RHG family (detailed in Additional file 3 table A). Detailed analysis of primary families and genes involved in apoptotic pathways follows.

**Table 1:** Apoptosis-related genes of *Bombyx mori*

Gene name	Gene holoname	ORF length(bp)	Extron number	**Chromosome No**.	Scoffold	Domain	Est		**Prober No**.	Ncbi E-value
							number	integrity		
*BmAcinus*	apoptotic chromatin condensation inducer in the nucleus	2442	4	4	nscaf2847	No domain	4	0	sw01266	2e-113
*BmAif*	apoptosis-inducing factor	1794	13	14	scaffold416	Pyr_redox superfamily domain	2	0	sw03811	3e-86
*BmAkt*	pbk	1410	8	18	nscaf2902	PH-like superfamily/PKc-like superfamily domain	2	0	sw17926 sw07695	0.0
*BmApaf-1*	apoptotic peptidase activating factor 1	4302	18	23	nscaf3015	CARD/WD40 superfamily domain	1	0	sw17785	4e-41
*BmApp*	amyloid precursor protein	2067	15	18	nscaf2902	A4_Extra superfamily/GBP_C domain	0	0	sw03656	0.0
*BmAsk1*	apoptosis signal-regulating kinase 1	4041	24	12	nscaf2993	S_TKc/SAM domain	0	0	sw19703	0.0
*BmAtf2*	activating transcription factor 2	1134	4	2	nscaf2053	ZnF_C2H2 domain	0	0	sw15358	4e-24
*BmBuffy*	B-cell lymphoma protein 2	879	6	4,7	nscaf2981 nscaf2983 nscaf3077	Bcl2family domain	3	0	sw21409	5e-23
*BmCaspase-1*	cysteine aspartic acic specific protease 1	882	1	10	nscaf2860	CASc domain	8	1	sw04417	0.0
*BmICE-5*	cysteine aspartic acic specific protease 3L	939	9	4	nscaf2847	CASc domain	10	1	sw22502	0.0
*BmICE-2*	cysteine aspartic acic specific protease 3S	855	8	4	nscaf2847	CASc domain	10	1	sw22502	1e-65
*BmICE*	interleukin converting enzyme	828	7	4	nscaf2847	CASc domain	10	1	sw22502	0.0
*BmDredd*	cysteine aspartic acic specific protease 8	1632	1	10	nscaf2855	CASc domain	1	0	Not found	0.0
*BmDroncL*	cysteine aspartic acic specific protease 9L	1233	7	10	nscaf2575	CARD/CASc domain	1	0	sw12316	2e-41
*BmDroncS*	cysteine aspartic acic specific protease 9S	552	5	10	nscaf2575	CARD/CASc domain	1	0	sw12316	1e-15
*BmCaspase-N*	cysteine aspartic acic specific protease New	1071	4	20	nscaf2795	CASc domain	2	0	sw04445	1e-28
*BmCdc2*	cell division cycle2	960	7	16	nscaf3062	Protein_kinase_domain	1	1	sw02944	0.0
*BmCreb*	cAMP response element binding protein	894	6	10	nscaf2859	pKID superfamily/bZIP_1 superfamily domain	4	1	sw14332	1e-145
*BmCyt c*	cytochrome c	327	3	3	nscaf2930	Cytochrom_C superfamily domain	45	1	sw15825	9e-58
*BmDapk*	death-associated protein kinase 1	1242	7	10	nscaf2855	PKc_like superfamily/S_TKc domain	0	0	sw11691	2e-68
*BmDaxx*	Fas death-domain associated protein	1785	6	3	nscaf2886	Daxx superfamily	3	0	sw18609	2e-12
*BmEndoG*	endonuclease G	807	6	20	nscaf2789	NUC superfamily domain	0	0	sw12629	2e-117
*BmErk*	extracellular-signal regulating kinase	990	7	10	nscaf2855	PKc_like superfamily	0	0	sw13977	0.0
*BmFadd*	fas-associated via death domain	666	2	22	nscaf1681	Death superfamily domain	1	0	Not found	0.89
*BmFkhr*	forkhead homolog in rhabdomyosarcoma	480	3	9	nscaf2890	FH superfamily domain	1	0	sw09818 sw11611	2e-44
*BmGas2*	growth arrest specific 2	1938	9	14	nscaf2948	CH superfamily/gas2 superfamily	5	0	sw12456 sw00851 sw05095	6e-106
*BmGsk3*	glycogen Synthase Kinase-3	945	7	18	nscaf2902	S_TKc/PKc_like superfamily/Pkinase domain	4	0	Not found	2e-159
*BmHtra2*	High temperature requirement A2	1740	11	26	nscaf2330	ROK/TrypP_SPc/PDZ superfamily domain	4	0	sw00567	1e-113
*BmIcad*	inhibitor of caspase-activated DNase	594	2	23	nscaf3015	CIDE-N superfamily domain	3	0	sw01954	0.0
*BmIap*	inhibitor of apoptosis protein	1041	2	23	nscaf3027	BIR superfamily domain	34	1	sw22890	0.0
*BmIap2*	inhibitor of apoptosis 2 protein	1686	9	23	nscaf3027	BIR superfamily domain	2	0	sw14099 sw01498	8e-97
*BmBruce*	----	12711	59	25	nscaf2823	BIR superfamily domain	5	0	sw10962	0.0
*BmSurvivin*	----	411	3	17	nscaf2766	BIR superfamily domain	16	0	sw16094	5e-32
*BmJnk*	c-Jun N-terminal kinase	1146	9	3	nscaf2883	PKc_like superfamily domain	2	0	sw14613	0.0
*Bmmkk7*	mitogen-activate protein kinase kinase7	1230	10	15	nscaf2888	PKc_like superfamily domain	0	0	sw18329	4e-138
*BmP53*	----	546	3	16	nscaf3063	P53-superfamily	1	0	sw20071	9e-14
*BmP70s6k*	p70 ribosomal protein S6 kinase	936	6	10	nscaf2859	PKc_like superfamily	2	0	sw00674	4e-90
*BmP90srk*	p90 ribosomal S6 kinase	2262	13	23	nscaf3015	S_TKc/PKc_like superfamily	0	0	sw22237	0.0
*BmPax6*	paired box gene 6	552	3	14	nscaf2953	pax superfamily	1	0	sw01861	2e-69
*BmParp*	poly ADP ribose polymerase	2898	18	22	nscaf2813	WGR/ADP ribosyl superfamily domain	6	0	sw03907	0.0
*Bmpdk*	pyruvate dehydrogenase kinase	1299	9	2	nscaf2623	HATPase_c superfamily domain	4	0	sw15422	0.0
*BmPi3k*	phosphatidylinositol 3-kinase	1197	4	12	nscaf2993	SH2 superfamily domain	1	0	sw21185	0.0
*BmPka*	protein kinase A	1062	1	23	nscaf3027	PKc_like superfamily	0	0	sw08116	0.0
*BmPkc*	protein kinase C	1590	8	8	nscaf463	C2/PKc_like superfamily	0	0	sw05322	0.0
*BmRaf*	----	2103	11	15	nscaf2888	VBQ/PKc_like superfamily	0	0	sw17787	0.0
*BmRas*	renin-angiotensin system	579	2	21	nscaf2868	P_loop_NTPase superfamily domain	1	1	sw09841	0.0
*BmReaper*	----	93	1	7	nscaf2983	IBM binding and GH3 motifs	2	1	Not found	
*BmRock1*	Rho kinase	4083	12	4	nscaf2589	PKc_like superfamily	0	0	sw12299	0.0
*BmSir2*	silent information regulator 2	1872	11	25	nscaf2823	ASC/SIR2 superfamily	2	0	sw10596	0.0
*BmStat*	signal transducer and activator of transcription	2178	21	11	nscaf2176	STAT_int/STAT_alpha/STAT_bind/SH2 superfamily domain	4	0	sw09865	0.0
*BmTak1*	TGF-beta-activated kinase 1	1197	7	26	nscaf3003	PP2Cc superfamily domain	2	0	sw14675	7e-87
*BmTNFSF5*	Tumor Necrosis Factor superfamily 5	648	3	5	nscaf2674	TNF superfamily	2	0	sw08247	0.23
*BmTNFSF13*	Tumor Necrosis Factor superfamily 13	492	3	5	nscaf2674	TNF superfamily	3	0	sw13698	3e-06
*BmTRAF3*	TNF-receptor-associated factor 3	1479	9	14	nscaf2953	MATH superfamily	1	0	sw04672	1e-43
*BmTRAF6*	TNF-receptor-associated factor 6	1080	6	13	nscaf1898	MATH superfamily	0	0	sw03276	2e-62

The complete and incomplete EST sequences are represented by 0 and 1, respectively. No domains are displayed when the BmAcinus is BLASTed with the database in NCBI, but the first sequence in the result is submitted by the partner of the Silkworm Institutes in Zhen Jiang.

**Table 2:** Comparision of the key families and genes involved in apoptosis in mammals, Drosophila, Nematode, Echinoids, silkworm and fish.

Species name Families/genes'name	**Mammal**^**a**^	**Fruit Flies**^**b**^	**Nematode**^**c**^	**Echinoids**^**d**^	**Silkworm**^**e**^	**Fish**^**f**^
TNFSF^A^	50	2	0	11	2	11
Caspase Family^B^	13	7	4	34	5	12
Bcl-2 Family^C^	about 20	2	3	10	1	13
Bir Domain^D^	7	4	2	7	4	5
RHG^E^	0	4 (each1)	0	0	1 (reaper)	0
P53^F^	1	1	1	1	1	1
Apaf-1^G^	1	1	1	1	1	1

In this study *Homo sapiens *represents mammals, *Drosophila melanogaster *represents fruit flies, *Caenorhabditis elegans *represents nematodes, *Strongylocentrotus purpuratus *represents Echinoids, *Bombyx mori *represents silkworms, and *Danio rerio *represents fish. The TNF family comprised of the ligands and the correspondent receptor. aA data are from [76-78]; aB data are from [79]; bB, cB, bC and cC data are from [65] and [6]; dA-dD and fA-fC data are from [80] and [81], respectively. The other data are the results of BLAST searches with the database in NCBI.

### Caspase family members in silkworm

**Figure 1:**
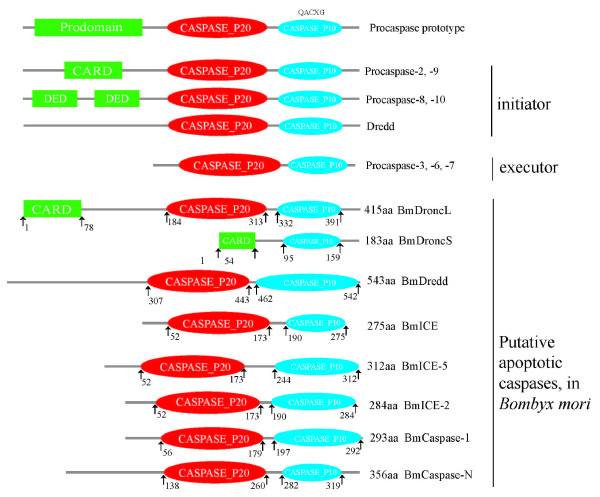
**Schematic diagram of putative caspase family members in *Bombyx mori***. A diagrammatic representation of the domain architecture of the initiator, executioner and putative apoptotic caspase proenzymes of the silkworm is shown here. The protein sequence "QACXG" is in the small subunit of all caspase family members; X is usually R or Q. The numbers are the start and end of these domains in the protein sequences.

Caspase are a family of cysteinyl aspartate proteinases with two main branches: the pro-inflammatory ICE-like subfamily, previously found only in vertebrates [62], and the apoptotic caspase subfamily. All caspases, normally present as inactive proenzymes in cells, have three different regions: N-terminal prodomains, a large catalytic domain (approximately 20 kD, known as P20) and a small catalytic domain (approximately 10 kD, known as P10). Based on the length of the prodomain, caspases are divided into two groups: class I (initiators), which have a relatively long prodomain, and class II (effectors), which have a short prodomain (Figure 1). Based on the N-terminal prodomain, the initiators can be divided further into two categories: one class containing a caspase recruitment domain (CARD), such as caspase-2 and -9 in mammals and DRONC in fruit flies, and the other possessing a death effector domain (DED), such as caspase-8 and -10 in mammals. The mammalian caspase-8 is replaced functionally by the *Drosophila *homologue DmDredd, while DmDredd does not have a DED in its N-terminal prodomain [63,64] (Figure 1).

**Figure 2:**
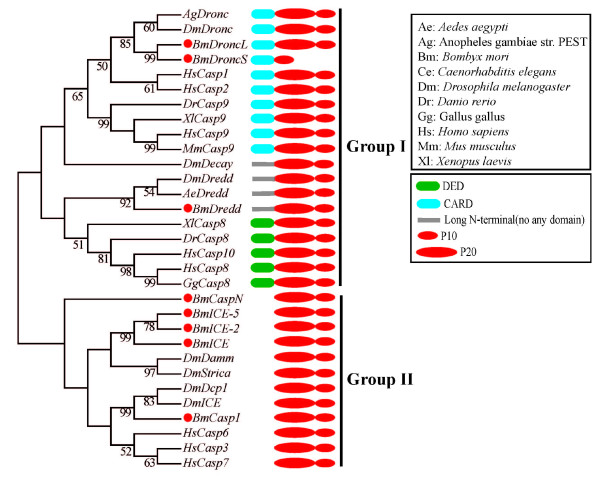
**The caspase family tree and domain architecture**. Multiple sequence alignments were performed using musle.exe. The neighbor-joining tree was produced using MEGA4.0. The putative caspase family members identified in *Bombyx mori *are marked with large red dots. The domain architectures and the full species names are listed in the black frame on the right.

Five caspase family homologs were cloned from the silkworm, including 2 initiators (*BmDredd *and *BmDronc*), and 3 effectors (*BmCaspase-1, BmICE, *and *BmCaspase-N*; Figure 2). The phylogenetic tree of caspase family members in silkworm and other species showed that all the intiator and effector homologs are clustered into group I and group II, respectively. The initiator caspases containing DED domain are clustered into group I, while others containing CARD domain and DmDecay into the other subgroup (Figure 2). The results reveal that the caspase family members are functionally conserved between species.

### Silkworm initiator caspase homologs *BmDronc *and *BmDredd*

Caspase-9 has a crucial role during apoptosis from nematodes to mammals. Recently, Dronc (*Drosophila *Nedd2-like caspase) homologs have been identified in the genomes of *Aedes aegypti *and *Anopheles gambiae *(GenBank Accession: 1278470) [65]. The mammalian *caspase-9 *homolog *BmDronc *was identified in silkworm, which has two splice variants with common translation initiation and termination sites, named *BmDroncL *(415 aa) and *BmDroncS *(183 aa) respectively, and verified by cloning from silkworm BmE cells (Figure 1). Only *BmDroncS *has a CARD domain and a small subunit, while *BmDroncL *has the typical caspase family domains (Figure 1). The sequence identities of silkworm *BmDroncL *with homologs from *Homo sapiens*, *Drosophila melanogaster*, and *Aedes aegypti *are 24%, 29% and 35%, respectively.

An ortholog of *Drosophila *Dredd was also found in the silkworm, named *BmDredd *(1950 bp, 543aa, figure 1), consistent with the data submitted to NCBI (GeneID: 100141428). The result of domain prediction revealed that BmDredd, AeDredd and DmDredd have long prodomains but no domains that mark them as initiator caspases (Figure 2), unlike mammalian caspase-8 with DEDs. The genetic relationship between caspases from silkworm, *Aedes aegypti *and *Drosophila *is much closer than with other species (Figure 2). The sequence identities and similarities of BmDredd with DmDredd and AeDredd are 28% and 45%, 27% and 45%, respectively.

### Silkworm effector caspases

*Bmcaspase-1 *is the first effector caspase reported in *Bombyx mori*. Pei and colleagues [51] identified *Bmcaspase-1 *(GeneBank Accession: AF448494) from *Bombyx mori *BmN cells. *Bmcaspase-1 *has only one exon, is 1291 bp long, coding for 293aa, and is located on chromosome 10 (Table 1). Bmcaspase-1 has the classic short prodomain, with the characteristic QACXG sequence in the large subunit (residues 56 - 179) and the small subunit (residues 197 - 292; Figure 1). From the evolutionary relationship, BmCaspase-1 clusters into the same group with DmICE and DmDcp-1 of *Drosophila melanoganster *(Figure 2). Duan and colleagues [52] identified *BmICE *(275 aa, GeneBank accession: AY88522) from the *Bombyx mori *integumentum, and Song and colleagues [53] have cloned *BmICE-2 *(284 aa) and *BmICE-5 *(312 aa; GeneBank accession numbers DQ360829 and DQ360830, respectively) according to the sequence submitted by Duan [52] from BmE cells. All these genes have the characteristic QRCAG sequence and the typical large/small subunit configuration of caspase family members (Figure 1). We aligned and analyzed the sequences of these three coding sequences with the silkworm 9× genome database, and the result reveals that *BmICE*, *BmICE-2 *and *BmICE-5 *have the same translation and termination sites (Additional file 4). *BmICE *has seven exons, *BmICE-2 *has eight exons and *BmICE-5 *has nine exons; the splicing differences occur after the seventh exon (Additional file 4).

Our results demonstrated that there is an additional caspase family member in *Bombyx mori*. Because it has not been reported in *Bombyx mori *previously, we named it *BmCaspase-New *(*BmCaspase-N*). *Bmcaspase-N *is 1071 bp long, coding for 356 aa, and possesses the characteristic structure of caspase effector subfamily members, including a short prodomain and CASc domain with the QACXG sequence. Phylogenetic analysis revealed that *Bmcaspase-N *clusters into the effector group (Figure 2). Thus we propose that *BmCaspase-N *belongs to the effector caspase subfamily.

### Bcl-2 family members in silkworms

Bcl-2 family members participate at a crucial point in apoptotic pathways. All members share at least one of four BH domains (BH1, BH2, BH3 or BH4). Tambunan and colleagues [49] identified BmP109 from samples obtained during silk gland histolysis, a stage of *Bombyx mori *metamorphosis. However, the function of BmP109 with all four conserved BH regions has not been confirmed in *Bombyx mori*.

**Figure 3:**
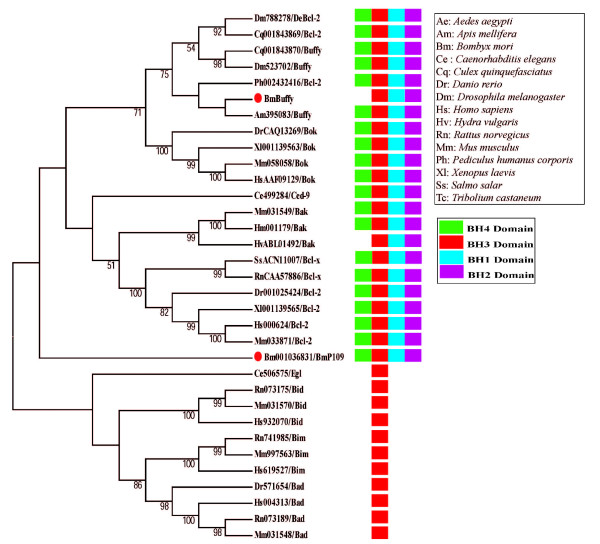
**Phylogenetic analysis of *Bombyx mori *Buffy with homologs in other species and their domain architectures**. Multiple sequence alignments were performed using musle.exe. The tree was produced using the neighbor-joining method. The putative Bcl-2 family members identified in *Bombyx mori *are marked with large red dots. The protein names use an abbreviation of species and gene names. The domain architectures and the full species names are listed in the black frame on the right.

We analyzed and cloned the other Bcl-2 family homolog *BmBuffy*, whose structure is more similar to *Buffy *of *Apis mellifera *and bcl-2 of *Pediculus humanus corporis *(Figure 3). BmBuffy lacks the BH4 domain. The completed *BmBuffy *cDNA is 1632 bp, coding for 292 aa, and the relative predicted molecular mass is 32.38 kDa. The sequence similarity and identity are 51% and 27%, respectively, compared with *DmBuffy*.

### BIR domain family members in silkworms

**Figure 4:**
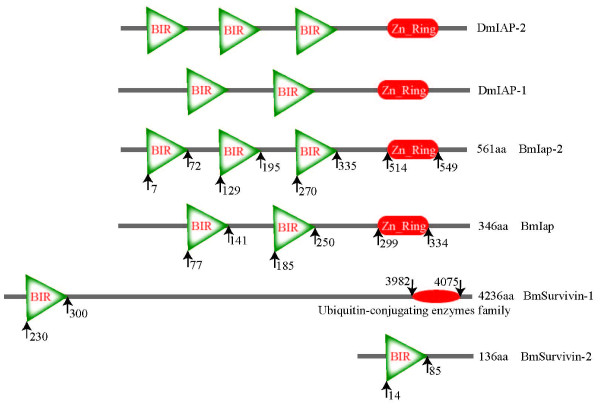
**Structures of the proteins in *Bombyx mori *predicted to contain BIR domains**. A diagrammatic representation of the domain architecture of the Bir domain members of the silkworm and the two IAPs in *Drosophila *is shown here. The numbers are the start and end location of the domains in the protein sequences.

The BIR domain is a unique structure originally identified in IAP proteins from baculoviruses. At least one BIR motif is essential for the antiapoptotic activity of IAP family members, but not all BIR-containing proteins (BIRPs) are IAPs [56]. We identified four proteins containing BIR domains in *Bombyx mori*, including two IAPs, one Bruce and one survivin. Huang and colleagues [50] cloned the first IAP family member *BmIAP *from *Bombyx mori *BmN cells. BmIAP is a specific inhibitor of mammalian caspase-9, but does not directly inhibit the downstream effector proteases caspase-3 and caspase-7. BmIAP inhibits apoptosis induced by Bax but not Fas *in vitro*. However, the function of BmIAP *in vivo *is not yet known. The other IAP family member *BmIAP2 *is located on the same chromosome as *BmIAP*, is 561 aa long and possesses three BIR domains and one Zn^2+^-finger domain. Compared with DIAP1 and DIAP2, BmIAP1 and BmIAP2 have two and three BIR domains, respectively, also (Figure 4). The BmBruce and survivin proteins each have one BIR domain, with a sequence consistent with the online BIR sequence (http://www.expasy.org/cgi-bin/nicedoc.pl?PS50143), and are 4236 aa and 136 aa long, respectively (Figure 4). Besides their size difference, BmBruce also has an ubiquitin-proteasome binding motif, which is homologous to *Drosophila *Bruce protein [66].

RHG family members in *Drosophila *contain the IAP-Binding-Motif (IBM) domain in their N-terminal, which binds to and removes the inhibitory activity of IAP [21,28,67] as well as their structural and functional homologs Smac/Diablo in mammals [68]. However, RHG family proteins connect many different signaling pathways, thereby having a central role in the regulation of programmed cell death in *Drosophila*, which is very different from other species, especially compared to mammalian Smac/Diablo[69]. Another IAP inhibitor, Htra2/Omi in mammals and its homolog protein DmHtra/Omi in *Drosophila*, have a function similar to Smac/Diablo. DmHtra2/Omi in *Drosophila *also has serine protease activity. Interestingly, reducing DmOmi expression by RNAi in the fly inhibits stress-induced apoptosis, while the neurodegeneration is increased in the Htra2/Omi knockout mouse [28]. The homolog *BmReaper*, an ortholog of Drosophila *reaper*, was found in silkworm[54]. *BmReaper *has both IBM and GH3 domain, which can bind to BmIAP and induce apoptosis in insect cells. The homolog *BmHtra2 *was also found in the silkworm and cloned (Additional file 2).

### TNFSF and their receptors in silkworm

**Figure 5:**
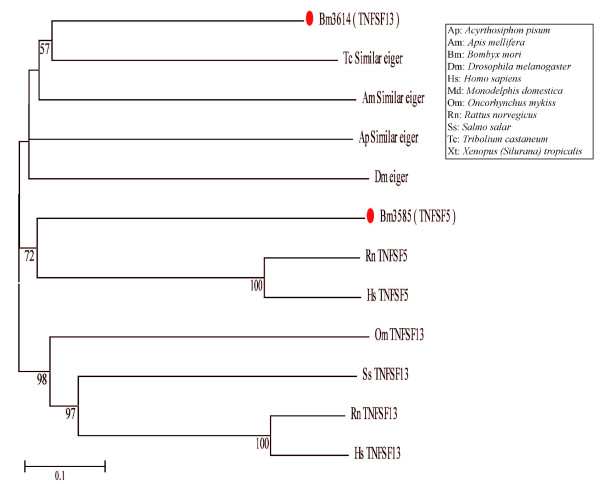
**Phylogenetic tree of the TNF superfamily**. This phylogenetic tree of the TNF superfamily was produced by neighbor-joining of multiple sequence alignment of TNF superfamily sequences from insect and the homologous sequences of the TNFSF from higher animals [including *Homo sapiens*, *Rattus norvegicus*, *Monodelphis domestica*, *Oncorhynchus mykiss*, *Salmo salar *and *Xenopus *(*Silurana*) *tropicalis*]. Because the silkworm TNFSF homologs were produced by alignment with TNFSF5 and TNFSF13, the TNFSF sequences used in this phylogenetic tree were the TNFSF5 and TNFSF13 in different species. The TNFSF members predicted in *Bombyx mori *are marked with large red dots. The protein names use an abbreviation of species and gene names. The full species names are listed in the black frame on the right.

TNF family ligands and their corresponding receptors (TNFR) have pivotal roles in many important physiological processes, such as host defense, inflammation, apoptosis, autoimmunity and immune system organogenesis. The TNF-related ligands are type II (intracellular N-terminus) transmembrane proteins containing a TNF homology domain (THD) at the extracellular C terminus. Protein sequences of 18 TNFSF ligands in mammals[70] and the TNF ligand Eiger in *Drosophila*[62] used as queries were aligned with the silkworm predicted protein database by BlastP. Two TNFSF members, Bm3585 and Bm3614, were identified. They are located on chromosome 5 (Table 1). Bm3585 and Bm3614 possess the typical THD as predicted (http://blast.ncbi.nlm.nih.gov/; Table 1), and demonstrated that potential TNF ligands are present in *Bombyx mori*. The phylogenetic tree of TNFSF between silkworm and other species show that Bm3614 and the insect Eiger homolog are in one cluster, while Bm3585 and TNFSF5 are classified close together, but the two TNF ligand homologs are evolutionarily distant (Figure 5), all of which indicate that a gene deletion or duplication event might had happened.

Using sequence information from 31 TNFR superfamily (TNFRSF) proteins[70] from mammals and one TNFRSF protein from insects, a search for possible TNF receptors was performed in *Bombyx mori*, but no match to the TNFR domains was found. However, many predicted proteins (mainly transmembrane proteins, fiber proteins, or mucous membrane proteins) possessed all the structural motifs, such as a cysteine-rich domain, Ca^2+^-binding site, and receptor/ligand interaction site (data not shown), but did not meet our criteria. However, homologs containing DDs (death domains) such as *BmDaxx *and *BmFadd*, were found in the silkworm.

### Expression profiles of apoptosis-related genes in silkworm

#### ESTs analysis

In order to detect the expression of the *Bombyx mori *apoptosis-related genes, we searched the silkworm dbEST database downloaded from GenBank using the putative coding sequences as queries. Forty apoptotic genes matched at least one EST. Nine genes had complete expressed sequence tags (ESTs), and the remaining genes had incomplete ESTs (Table 1).

#### Microarray-based gene expression profiles in different development stages

**Figure 6:**
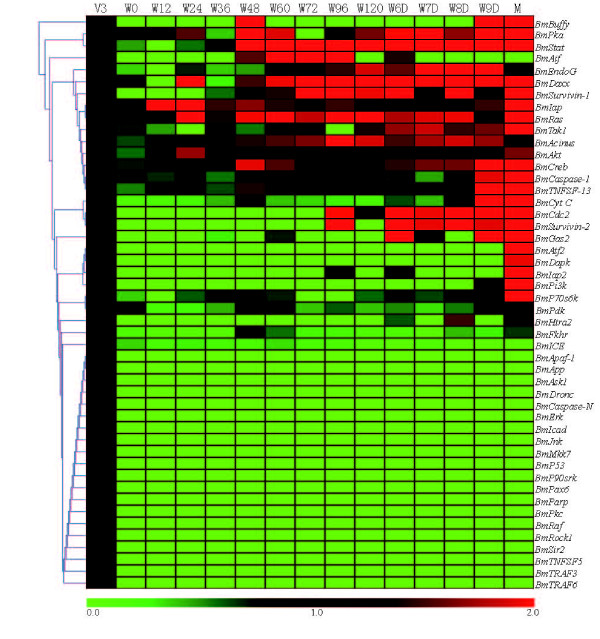
**Microarray analysis of the expression of the putative silkworm apoptosis-related genes during different growth stages**. The expression levels of all the genes in different stages compare to their expression level in the 3^th ^day of fifth instar. V3: 3^th ^of fifth instar; W0: 0 h after wandering; W12: 12 h after wandering; W24: 24 h after wandering; W36: 36 h after wandering; W48: 48 h after wandering; W60: 60 h after wandering; W72: 72 h after wandering; W96: 96 h after wandering; W120: 120 h after wandering; W6D: 6^th ^day after wandering; W7D: 7^th ^day after wandering; W8D: 8^th ^day after wandering; W9D: 9^th ^after wandering; M: silk moth. Red is a ratio ≥1, green is a ratio ≤1, and black is a ratio = 1.

To analyze the expression of the silkworm apoptosis-related genes in different developmental stages according the chips, a BlastN alignment was performed using the silkworm different developmental stage database. The results indicated that all the apoptosis-related genes contained at least one oligonucleotide probes except *BmDredd, BmFadd, BmGsk3 *(Table 1), for which no probe was found. Only 26 apoptosis-genes show higher expression than in the 3rd day of the fifth instar, when almost all genes expressed in the silkworm are present (Figure 6). The results revealed that the expressions of apoptosis-genes are relative low in silkworm.

#### The identification of apoptosis-related genes in silkworm

**Figure 7:**
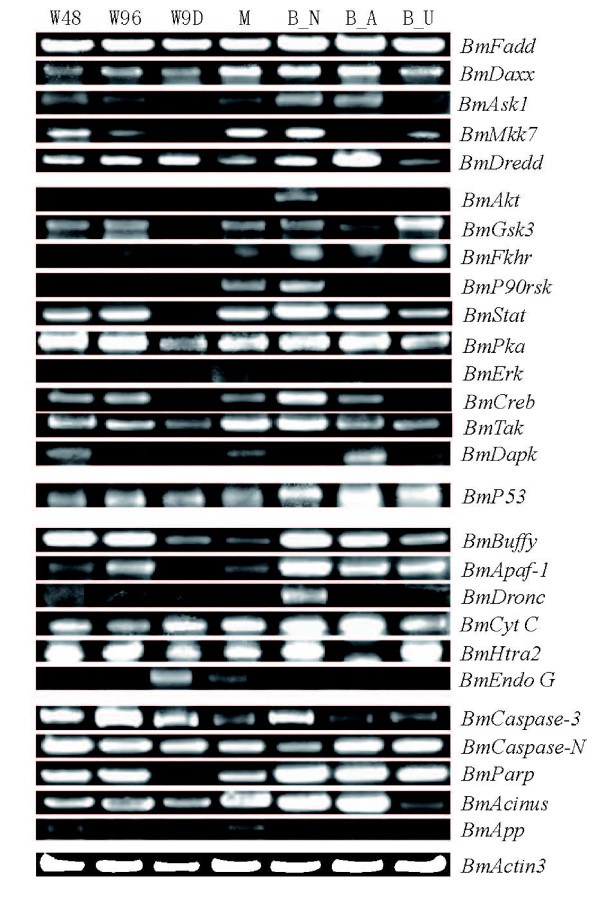
**RT-PCR analysis of the expression of the putative silkworm apoptosis-related genes during different growth stages and in BmE cells**. Total RNA was isolated as described in the Methods section and analyzed by RT-PCR. W48: about 48 h after wandering; W96: about 96 h after wandering; W9D: about 9^th ^day after wandering; M: silk moth; B_N: normal BmE cells; B_A: BmE cells exposed to actinomycin D; B_U: BmE cells exposed to UV irradiation.

To test the expression of the apoptosis genes, we designed primers for 32 genes according to their predicted DNA sequences (Additional file 2) and carried out RT-PCR using the cDNA isolated from the different development stages of silkworm and BmE cells exposed to different stressors as described in the Methods section. Twenty three of all apoptosis-related genes tested were cloned and sequenced, five apoptosis-related genes were detected by RT-PCR but not sequenced successfully, although the PCR product sizes were consistent with the predicted sizes, and four silkworm apoptosis-related genes were not detected (Additional file 2). Overall, the expression of these genes, except a few, was relatively much lower than *BmActin3 *expression (Figure 7). The results show that most of the key apoptosis-genes in silkworm are expressed, which revealed that these potential apoptosis-genes are present in *Bombyx mori*.

## Discussion

### The silkworm apoptosis-related genes

In this study, we identified and cloned 52 silkworm apoptosis-related genes, including homologs of almost all the key genes involved in apoptosis pathways in other species. The fact that the BH3-only subfamily only existed in vertebrates, while the RHG family is found only in insects, reveals conservation within species and the variability among the species, although their functional homologs exist in mammals. Moreover, the main families of apoptosis-related genes exist in all model organisms, but the gene numbers in some species (such as the caspase family in *Strongylocentrotus purpuratus*) are much higher, indicating that expansion might have occurred in these species, most likely due to environmental stress.

The key genes involved in apoptosis pathways in *Bombyx mori *are described in detail in Table 1. Interestingly, TNFSF members containing DDs, caspase family members involved in inflammation, and the BH3-only Bcl-2 family members are not found in *Bombyx mori*. However, the silkworm not only has an insect Eiger homolog *Bm3614*, but also has *Bm3585*, which is similar to mammalian TNFSF5, neither of which have been reported in either *Drosophila *or mosquito. These results suggest that some genes may be lost in evolution. The new putative effector caspase *Bmcaspase-N *was found in silkworms, but not in mosquitoes [71] or *Drosophila*, suggesting that gene expansion occurred in *Bombyx mori *after the insect diverged from the common ancestor. For example, since *BmDroncS *only has a CARD and a small subunit that lacking the core active site, it may act as a caspase-like decoy molecule [72]. Furthermore, the phylogenetic analysis of caspase family members in *Bombyx mori *with those involved in apoptotic pathways in other species shows that caspase-8 homologs lacking DED domains in insects are clustered into the same class, which suggests that the caspase-8 homology gene mutation might have occurred after divergence of animals and insects. In contrast, the presence of all caspase-9 homologs in the same class suggests caspase-9 is highly conserved from insects to mammals (Figure 2). In addition, BmBruce has an ubiquitin-proteasome binding motif, which suggests that the ubiquitin-proteasome pathway may be present in *Bombyx mori*, as in *Drosophila *[69].

### The possible apoptosis pathways of *Bombyx mori*

The apoptosis-related factors identified in silkworms cover almost all the critical junctions in the apoptosis pathways of other model organisms. Although Fas and its receptor were not found, we found some proteins predicted containing cysteine regions, Ca^2+ ^binding sites, or the typical receptor/ligand interaction sites of TNFRSF members (data not shown), downstream genes such as *BmTraf *family members and *BmFadd*, which contain DDs, and *BmDredd*. All these results suggest that the death receptor pathway may be present in *Bombyx mori*. We hypothesize that the epidermal growth factor pathway also exists in *Bombyx mori*, because homologs of the mammalian members of this pathway were cloned and identified in *Bombyx mori*, including *BmRaf*, *BmRas*, *BmPka*, *BmPkc*, *BmErk*, *BmPi3k*, *BmStat*, *BmAkt*, *BmGsk3*, and *BmFkhr*. The *Bombyx mori *homologs of *Cyt C*, *Apaf-1*, *Caspase-9*, *Aif*, *Endo G*, and *Htra2 *were identified and characterized in *Bombyx mori*, and Kumarswamy and colleagues [29] and the Pan group [30] have demonstrated cytochrome C release into the cytoplasm in stress-induced apoptotic Sf-9 and BmE cells. Therefore, the mitochondrial apoptotic pathway may be functional in *Bombyx mori*. Furthermore, the *DmReaper *orthologs found in *Bombyx mori *indicate that this apoptotic gene is conserved between species. In addition, key genes in the DNA damage response pathway, like *BmP53 *and *BmSir2*, are also identified, so we hypothesize that the DNA damage pathway is also functional in *Bombyx mori*. In conclusion, the intrinsic and extrinsic pathways described in other models may potentially exist in *Bombyx mori*.

In 1965 silkworm hemolymph was used as an additional agent in cell culture *in vitro *[73]. Rhee and colleagues confirmed that silkworm hemolymph inhibited cell apoptosis not only in a baculovirus-induced insect cell system [35] but also in a human cell system [36]. In 2002, this group reported that they isolated and characterized an apoptosis-inhibiting hemolymph component [34]. Later they constructed a recombinant vector to express the protein and purify it *in vitro*, and confirmed this protein is one of the 30K proteins isolated from silkworm hemolymph used to minimize cell death. They speculated that the 30Kc6 protein inhibits the apoptosis by involvement upstream of caspase3 activation [37,38]. Therefore, there might be differences between *Bombyx mori *and other models in the regulation of apoptosis. Questions remain as to the precise regulation of apoptosis in *Bombyx mori*: for example, the central role of *BmReaper*, as well its homolog in *Drosophila*, and whether *BmCytC *is released from the mitochondria as in mammals. Also, the BH3-only Bcl-2 family members that link the two primary apoptotic pathways are not found in *Bombyx mori *and have not been reported in insects. Identification of a surrogate protein that performs the same function would provide great insight into apoptosis in the silkworm.

## Conclusions

Biochemical evidence and comparative genomic analyses with mammals and other organisms show that many apoptosis-related gene homologs are present in *Bombyx mori*, and suggest that the typical apoptotic pathways exist in *Bombyx mori*. The identification of these new genes in *Bombyx mori *further supports the universality of apoptotic mechanisms. The data in this study provide an overview for putative apoptosis-related genes in *Bombyx mori*, which should contribute to mechanistic studies of apoptosis in *Bombyx mori *in the future.

## Methods

### Cell line and *Bombyx mori*

The BmE (*Bombyx mori *Embryo) cell line BmE-SWU1[74] was cultured in Grace medium containing 10% fetal bovine serum (FBS) at 27°C in an incubator. The *Bombyx mori *DaZao strain larvae were bred with fresh mulberry leaves at 25°C with a 12 h:12 h photoperiod.

### Identification of silkworm apoptosis-related genes

The databases used for the *Bombyx mori *genomic information include *Bombyx mori *9x genomic sequencing database, *Bombyx mori *EST database, CDS database, and predicted protein database (all found through http://silkworm.swu.edu.cn/silkdb/). The nucleotide and protein sequences of apoptosis-related homolog of different species (including *Bombyx mori*, *Drosophila melanogaster*, *Caenorhabditis elegans*, *Homo sapiens*) were obtained from the NCBI database (http://www.ncbi.nlm.nih.gov) (described in detail in Additional file 3 Table B).

For the comparison analysis, the gene sequences, mRNA sequences, and protein sequences of apoptosis-related gene homologs in various sepecies were downloaded from NCBI. Three methods were used as follows:

1. The protein sequences of apoptosis-related genes as queries were aligned with the predicted protein database by the BlastP program using amino acid sequence homology and an E-value of 0 (to account for the large differences between species). Predicted silkworm genes in the comparison results were selected to compare with the NCBI protein database (http://blast.ncbi.nlm.nih.gov/Blast.cgi) for further confirmation. If the predicted gene contained the same domains as its homolog and the first genes in the alignment result is the homolog in other species, then the predicted gene was considered a homolog in silkworm.

2. The sequences of the conserved domains of the gene intercepted were used as queries to perform BlastP searches against the silkworm predicted protein database and TBlastN searches against the silkworm 9x genome sequence using an E-value of 0. The identification is the same as described above. (TNFSF family members, BH-3 only subfamily members and RHG family members were analyzed using this method.)

3. Apoptosis-related genes not found in the silkworm database, were searched against the silkworm EST database by TblastN program using an E-value of 0. A method of cloning electronically was used, and we confirmed the result as above. (*BmBuffy *was checked using this method)

Finally, to acquire detailed information about the predicted gene, all the putative apoptosis-related genes in *Bombyx mori *were aligned with the 9x silkworm genomic database, EST database, and the microarray chip databases for different developmental stages using the BlastN program.

### Domain prediction, multiple sequence alignments and phylogenetic tree construction

All the domains of the putative apoptosis-related genes of the silkworm *Bombyx mori *are predicted (http://blast.ncbi.nlm.nih.gov/Blast.cgi) or (http://www.expasy.org/prosite/). Multiple sequence alignments were carried out using Clustal W or Muscle.exe programs. Phylogenetic analyses were performed using MEGA version 4.0[75].

### Treatments and RNA extraction

BmE cells were exposed to 200 ng/ml actinomycin D for 12 h or irradiated for 70 s with 30 J/m^2 ^UV, and then cultured normally for another 12 h. Larva [approximately 48 hours after wandering (spinning just finished)], pupa [approximately 96 hours after wandering (newly formed) and on approximately the 9th day after wandering (the day before becoming a moth)], and the adult moth were collected, and frozen immediately in liquid nitrogen. Total mRNA was extracted using Trizol reagent (Invitrogen, USA), and DNA contamination in the mRNA samples was digested with RNase-free DNase I (Takara, Japan). The concentration of RNA was calculated by spectrophotometry (Gene Spec V; HITACHI, Japan). The first strand of cDNA was synthesized from 1 μg mRNA using M-MLV Reverse Transcriptase following the manufacturer's instructions (Promega, USA).

### Verification of the putative silkworm apoptosis-related genes

The PCR primers were designed based on the coding sequences of the putative silkworm apoptosis-related genes identified by the bio-informatics analysis (Additional file 2). Silkworm cytoplasmic actin A3 gene (forward primer: 5'-AAC ACC CCG TCC TGC TCA CTG-3'; reverse primer: 5'-GGG CGA GAC GTG TGA TTT CCT-3') was used as an internal control. PCR amplification was performed in a total reaction volume of 25 μl, containing normalized cDNA (6 μg cDNA except *BmActin3 *using 3 μg cDNA), 15 pmol of each primer (1 μl), 2 mM MgCl_2 _(2 μl), 0.25 mM dNTP (2 μl), 1× buffer (2.5 μl), 2.5 units of Taq DNA polymerase and distilled deionized H_2_O. PCR was performed as follows: initial denaturation at 94°C for 3 min, followed by 25 cycles of 30 s each at 94°C, 1 min annealing (at the temperatures listed in Additional file 2), 1-3 min extension at 72°C (the time depends on the length of the gene), and a final extension at 72°C for 10 min. The amplification products were analyzed on 1% agarose gels, and sequenced and confirmed by the Ying Jun Company and Bio-engineering (Shanghai, China).

### Analysis of apoptosis-related genes of different developmental stages

More than 184201 ESTs from *Bombyx mori *are available in the NCBI database. To search transcripts for individual apoptosis-related genes, a BlastN search was conducted against the silkworm EST database. The putative coding sequences were used as queries. A 95% or greater identity and minimum cut-off E-value (less than or equal to e-20) were employed to discriminate between duplicated genes. Microarray data analysis was performed as described by Xia and colleagues [59].

## Authors' contributions

JYZ and MHP made the study design, the data collection and analysis. JYZ drafted the manuscript. MHP revised the manuscript. ZYS, SJH and ZSY did partial data analysis. DL and DHZ helped to perform the experiment of RT-PCR. CL conceived of the study, and participated in its design and coordination. All authors read and approved the final manuscript.

## Supplementary Material

Additional file 1 **The IDs of genes identified in the genomic database of silkworm.** Table A - The IDs of Genes submitted previously. All these genes submitted to NCBI and their GIs were listed in the table. And these genes were submitted by us in the table A, while that have been submitted by others in the table B. "+" represents reported genes, while "-" represents genes not reported but only submitted to NCBI (see the comments for table B). **Table B - The IDs of new identified Genes**. Click here for file

Additional file 2 **The primers and annealing temperatures of apoptosis-related genes in the silkworm**. Genes cloned and sequenced successfully are labeled "sequencing success," while genes cloned but not sequenced successfully are labeled "PCR success." Those genes neither cloned nor sequenced successfully are labeled "-". Click here for file

Additional file 3 **The apoptosis-related genes not searched in silkworm database and sequences aligned as queries.** Table A - The main genes homology not searched in silkworm were listed in the table. Table B - The sequences as queries. Because of limited space in the manuscript, partial genes' sequences in various species are displayed. The silkworm apoptotis-related genes and their protein accession included in NCBI are presented in red and bolded Click here for file

Additional file 4 **Three different splice variants of *BmICE *in *Bombyx mori***. 1, 2 and 3 are the splice variants of BmICE, named BmICE, BmICE-5 and BmICE-2 respectively. Click here for file
