# Supplementary material for: The genomic underpinnings of apoptosis in the silkworm, Bombyx mori
Source: BMC Genomics. 2010 Oct 31;11:611. doi: 10.1186/1471-2164-11-611 (PMC3091752; doi:10.1186/1471-2164-11-611)
Supplement: Additional file 1 — The IDs of genes identified in the genomic database of silkworm. Table A - The IDs of Genes submitted previously. All these genes submitted to NCBI and their GIs were listed in the table. And these genes were submitted by us in the table A, while that have been submitted by others in the table B. "+" represents reported genes, while "-" represents genes not reported but only submitted to NCBI (see the comments for table B). Table B - The IDs of new identified Genes. [file 1471-2164-11-611-S1.DOC]

| Gene name | | Genes ID | Comments | |
| --- | --- | --- | --- | --- |
| *BmAkt* | | 163962993 | **-** | |
| *BmCaspase-1* | | 112983104 | **+** | |
| *BmICE* | *BmICE* | 112983811 | **+** | |
|  | *BmICE-2* | 86371761 | **+** | |
|  | *BmICE-5* | 164448658 | **+** | |
| *BmCdc2* | | 112984382 | | **+** |
| *BmCyt C* | | 194303591 | | **+** |
| *BmDredd* | | 168823407 | | **-** |
| *BmErk* | | 112982894 | | **+** |
| *BmICAD* | | 168823417 | | **+** |
| *BmIAP* | | 14248546 | | **+** |
| *BmJnk* | | 154816104 | | **+** |
| *BmPdk* | | 167860162 | | **-** |
| *BmPka* | | 153792580 | | **-** |
| *BmPkc* | | 112983438 | | **+** |
| *BmRas* | | 112983398 | | **-** |
| *BmReaper* | | 261599026 | | **+** |
| *BmSir2* | | 112983108 | | **-** |
| *BmStat* | | 255652895 | | **-** |

| Gene name | Bankit NO. | GeneBank Accession |
| --- | --- | --- |
| *BmBuffy* | 1247733 | GQ426279 |
| *BmDroncL* | 1247749 | GQ426280 |
| *BmDroncS* | 1247753 | GQ426281 |
| *BmAcinus* | 1247759 | GQ426282 |
| *BmAif* | 1247766 | GQ426283 |
| *BmApp* | 1247769 | GQ426284 |
| *BmAsk1* | 1247775 | GQ426285 |
| *BmAtf2* | 1247814 | GQ426286 |
| *BmCaspase-N* | 1247853 | GQ426287 |
| *BmCreb* | 1247857 | GQ426288 |
| *BmDapk* | 1247862 | GQ426289 |
| *BmDaxx* | 1247865 | GQ426290 |
| *BmEndo G* | 1247870 | GQ426291 |
| *BmFadd* | 1247879 | GQ426292 |
| *BmFkhr* | 1247883 | GQ426293 |
| *BmGas2* | 1247885 | GQ426294 |
| *BmGsk3* | 1247891 | GQ426295 |
| *BmHtra2* | 1247894 | GQ426296 |
| *BmIap2* | 1247899 | GQ426297 |
| *BmSurvivin-1* | 1247913 | GQ426298 |
| *BmSurvivin-2* | 1247917 | GQ426299 |
| *BmMkk7* | 1247920 | GQ426300 |
| *BmP53* | 1247927 | GQ426301 |
| *BmPax6* | 1247952 | GQ426302 |
| *BmParp* | 1247955 | GQ426303 |
| *BmPi3k* | 1247960 | GQ426304 |
| *BmRaf* | 1247967 | GQ426305 |
| *BmTak1* | 1247968 | GQ426306 |
| *BmTNFSF-5* | 1247980 | GQ426307 |
| *BmTNFSF-13* | 1247984 | GQ426308 |
| *BmTraf-3* | 1247985 | GQ426309 |
| *BmTraf-6* | 1247990 | GQ426310 |
| *BmP90srk* | 1248906 | GQ426311 |
| *BmApaf-1* | 1384435 | HQ179968 |
| *BmP70S6K* | 1384445 | HQ179969 |
| *BmRock1* | 1384460 | HQ179970 |
