# Supplementary material for: The genomic underpinnings of apoptosis in the silkworm, Bombyx mori
Source: BMC Genomics. 2010 Oct 31;11:611. doi: 10.1186/1471-2164-11-611 (PMC3091752; doi:10.1186/1471-2164-11-611)
Supplement: Additional file 2 — The primers and annealing temperatures of apoptosis-related genes in the silkworm. Genes cloned and sequenced successfully are labeled "sequencing success," while genes cloned but not sequenced successfully are labeled "PCR success." Those genes neither cloned nor sequenced successfully are labeled "-". [file 1471-2164-11-611-S2.DOC]

| **Gene name** | **Forward primer(5'-3')** | **Reverse primer (5'-3')** | **Annealing temperature (℃)** | **ORF length（bp）** | | **Length cloned（bp）** | **Comments** |
| --- | --- | --- | --- | --- | --- | --- | --- |
| ***BmAcnius*** | AAACCACCACAAACTCCCGC | TCCCGAAGCCAATCCTCAAC | 59 | 2442 | | 1952 | Pcr success |
| ***BmAif*** | CGAGGCTCAAAAGTCCATAC | ACCATAGAGAAATCGTCCAAG | 56 | 1794 | | 1236 | - |
| ***BmAkt*** | GTGAGAAGGGAACGGGAAAG | TGTGAGAGAGGGCTGAATGC | 59 | 1410 | | 876 | sequencing success |
| ***Bmapaf-1*** | GGTTTGCTCGTAATGGAC | CAGGACCAGTGGAGGCT | 57 | 4302 | | 1189 | sequencing success |
| ***BmApp*** | GACCACGAAACCGCATAACTG | TTCCGACCATTCCTTCATCAC | 58 | 2067 | | 907 | Pcr success |
| ***BmAsk1*** | TTCAGCCCAACGAGTATGCGG | CCTTTGCCGAGCACCACGC | 62 | 4041 | | 805 | sequencing success |
| ***BmAtf2*** | TTTACAAAATGACAACCCCTT | GTCCAAATCACTCTGCTTCTC | 52 | 1134 | | 895 | Pcr success |
| ***BmBuffy*** | TTCCGACCAGATAACATTG | TTAGTCCATTCCATCCTCC | 57 | 879 | | 988 | sequencing success |
| ***BmIce*** | ATGGAGGAGACTTCGAACGAT | CTACTTTTCAAACATAAATAACTTAG | 57 | 852 | | 852 | sequencing success |
| ***BmDredd*** | AGTGACAGAAATGCTTGGAAC | AAATGGGAACCTGAGGATG | 55 | 1632 | | 1391 | sequencing success |
| ***BmDronc*** | TGAGATGGGACACTGGGATTTGGT | CTGGGAGGGTGGAGTTAGCGATG | 50 | 1233 | | 1438 | sequencing success |
| ***BmCaspase-N*** | AACAAAGAACAAGCGTAAGAAAT | TGAGCGTTGAAGTAATAACCG | 55 | 1425 | | 945 | sequencing success |
| ***BmCreb*** | TCTACCCAGTCAGGCTCCC | ACTCCTTTTTCTTTCTGCGG | 55 | 894 | | 659 | sequencing success |
| ***BmCyt C*** | CGTTGTCTCGATTAAGTGTCTA | GATTGTGTTGTGATTTGGAA | 47 | 540 | | 570 | sequencing success |
| ***BmDapk*** | CTCGTTCAGAAGTGGCTATCG | GTGCTTCAGCGGCTTGTC | 59 | 1242 | | 815 | sequencing success |
| ***BmDaxx*** | AGTTTGGGCTGGAATGCTG | TAAAGGACTGATAGGTCTACGGG | 57 | 1785 | | 518 | sequencing success |
| ***BmEndog*** | TACCAGCATTGCCTGTGTTC | GATTTACCGTCGGATTCTTTC | 56 | 807 | | 541 | sequencing success |
| ***BmFadd*** | CTCCAAGACACGGTGAGATT | TCCAAAGCATCACAAAGAAC | 53 | 666 | | 547 | sequencing success |
| ***BmFkhr*** | TCAGGGAGGCGGCGGTTAC | CCCCTTTGTCTTTGAAATATGGCAC | 55 | 480 | | 398 | sequencing success |
| ***BmGas2*** | GAGTCCTGAACACAAACCCT | ATGAAGACATTCCTTCCTGC | 55 | 1938 | | 1062 | - |
| ***BmGsk3*** | GCTCAAAGGTGACGACTG | ACCAAATAGCGGATTCTTAC | 53 | 945 | | 886 | Pcr success |
| ***BmHtra2*** | TGCGGTGGCTGGGGACATTTG | CGTCTCTCGGTAGGTCGTAGT | 61 | 1740 | | 529 | sequencing success |
| ***BmMkk7*** | TTGGCAGTGGTACATGCGGTC | CGACTTCACGACGAACGGG | 62 | 1230 | | 773 | sequencing success |
| ***BmP53*** | GGGCAATACAACTTCAGCGT | GGTGAAGATGACGGCTATGG | 57 | 546 | | 474 | sequencing success |
| ***BmP90srk*** | CGGCAAACAACCTTCCGATAG | CGCACCACCGAGAATGAACC | 60 | 2262 | | 1300 | sequencing success |
| ***BmParp*** | AACAAGAGAGCCGCCCCAG | GGTCCACAGCGTTGCCGTC | 61 | 2898 | | 1261 | Pcr success |
| ***BmPka*** | AACAATGCTGCCACCGCT | GCCGCCATCTCATACACG | 57 | 1062 | | 701 | sequencing success |
| ***BmRaf*** | ACACTCTAACTCGTCAGCGGA | GAGGTATATGATGGGGAAGGG | 59 | 2103 | | 1061 | sequencing success |
| ***BmRock1*** | CTTGTATGAGAATGGGACT | TCTGTCTGTAATCAACGCT | 53 | | 4083 | 1557 | - |
| ***BmSir2*** | TGTGTGGCAGAAACAGCGG | CATCTGGAACCTCTCGGGC | 61 | | 1872 | 1456 | - |
| ***BmStat*** | TTTCCCGATTGAAGTGAG | GGACTATGACCACAACCG | 52 | | 2178 | 1442 | sequencing success |
| ***BmTak*** | TGGAGTATTTGAGCCACAC | ATCTCATCTAAGAGCGTGC | 55 | | 1197 | 987 | sequencing success |
| ***BmActin3*** | AACACCCCGTCCTGCTCACTG | GGGCGAGACGTGTGATTTCCT | 53 | | about 750 | 750 | control |
