# Supplementary material for: The genomic underpinnings of apoptosis in the silkworm, Bombyx mori
Source: BMC Genomics. 2010 Oct 31;11:611. doi: 10.1186/1471-2164-11-611 (PMC3091752; doi:10.1186/1471-2164-11-611)
Supplement: Additional file 3 — The apoptosis-related genes not searched in silkworm database and sequences aligned as queries. Table A - The main genes homology not searched in silkworm were listed in the table. Table B - The sequences as queries. Because of limited space in the manuscript, partial genes' sequences in various species are displayed. The silkworm apoptotis-related genes and their protein accession included in NCBI are presented in red and bolded [file 1471-2164-11-611-S3.DOC]

| No. | Gene names |
| --- | --- |
| 1 | TNFSF(1-4,6-12,14-31) |
| 2 | TNFRSF(1-18) |
| 3 | Caspase family (2, 4,5,6,7,10,11,12,13,14,15) |
| 4 | RHG family (Hid, Grim, Sickle) |
| 5 | Bcl-2 family (Bcl-xl, Bcl-2, Bak,Bad,Bax,Bim,Bid,Pona) |
| 6 | Traf family (1, 2, 4,5) |
| 7 | TRADD |
| 8 | Smac |
| 9 | Raidd |
| 10 | NF-κB |

| **Gene name** | **Other species name** | **Corresponding genes’ holoname** | | | **Protein accession** |
| --- | --- | --- | --- | --- | --- |
| **Death Receptor Family** | | | | | |
| *BmTNFSF-5* | *Bos Taurus* | CD40 ligand | | | NP_777049 |
|  | *Mus musculus* | CD40 ligand | | | NP_035746 |
|  | *Pan troglodytes* | similar to TRAP | | | XP_529175 |
|  | *Canis lupus familiaris* | CD40 ligand | | | NP_001002981 |
|  | *Sus scrofa* | CD40 ligand | | | NP_999291 |
|  | *Gallus gallus* | CD40 ligand | | | NP_989996 |
|  | *Felis catus* | CD40 ligand | | | NP_001009298 |
|  | *Homo sapiens* | CD40 ligand | | | NP_000065 |
|  | *Rattus norvegicus* | CD40 ligand | | | NP_445805 |
| *BmTNFSF13* | *Homo sapiens* | TNFSP13 α | | | NP_003799 |
|  | *Homo sapiens* | TNFSP13 β | | | NP_742084 |
|  | *Homo sapiens* | TNFSP13γ | | | NP_742085 |
|  | *Mus musculus* | tumor necrosis factor (ligand) superfamily, member 13 | | | NP_076006 |
|  | *Oryctolagus cuniculus* | tumor necrosis factor (ligand) superfamily, member 13 | | | NP_001099146 |
|  | *Bos taurus* | tumor necrosis factor (ligand) superfamily, member 13 | | | NP_001099146 |
|  | *Rattus norvegicus* | tumor necrosis factor (ligand) superfamily, member 13 | | | NP_001009623 |
|  | *Pan troglodytes* | Similar to TWE-PRIL isoform 2 | | | XP_001171420 |
|  | *Pan troglodytes* | similar to TWEAK isoform 3 | | | XP_001171435 |
|  | *Pan troglodytes* | TNFSP13 isoform 4 | | | XP_001171471 |
|  | *Pan troglodytes* | TNFSP13 isoform 1 | | | XP_001171401 |
|  | *Canis familiaris* | similar to TNFSP13 isoform 2 | | | XP_849700 |
|  | *Canis familiaris* | similar to TNFSP13 isoform 3 | | | XP_859508 |
|  | *Canis familiaris* | similar to TNFSP13 isoform 1 | | | XP_536622 |
| *BmFADD* | *Homo sapiens* | Fas-associated via death domain | | | [NP_003815](http://www.ncbi.nlm.nih.gov/entrez/viewer.fcgi?val=NP_003815.1) |
|  | *Mus musculus* | Fas-associated via death domain | | | NP_034305 |
|  | *Xenopus laevis* | Fas-associating death domain-containing protein | | | [NP_001089017](http://www.ncbi.nlm.nih.gov/entrez/viewer.fcgi?val=NP_001089017.1) |
|  | *Sus scrofa* | Fas-associated via death domain | | | [NP_001026967](http://www.ncbi.nlm.nih.gov/entrez/viewer.fcgi?val=NP_001026967.1) |
|  | *Bos taurus* | Fas-associated via death domain | | | [NP_001007817](http://www.ncbi.nlm.nih.gov/entrez/viewer.fcgi?val=NP_001007817.1) |
|  | *Gallus gallus* | similar to Fas-associating death domain-containing protein, partial | | | XP_421073 |
|  | *Rattus norvegicus* | Fas (TNFRSF6)-associated via death domain | | | NP_690920 |
|  | *Bombyx mori* | Fas-associated via death domain | | | GQ426292***** |
| *BmDAXX* | *Homo sapiens* | death-associated protein 6 | | | [NP_001135441](http://www.ncbi.nlm.nih.gov/entrez/viewer.fcgi?val=NP_001135441.1) |
|  | *Drosophila melanogaster* | Daxx-like protein CG9537-PA | | | [NP_609035](http://www.ncbi.nlm.nih.gov/entrez/viewer.fcgi?val=NP_609035.2) |
|  | *Mus musculus* | Fas death domain-associated protein | | | [NP_031855](http://www.ncbi.nlm.nih.gov/entrez/viewer.fcgi?val=NP_031855.3) |
|  | *Bos taurus* | death-associated protein 6 | | | [NP_001068623](http://www.ncbi.nlm.nih.gov/entrez/viewer.fcgi?val=NP_001068623.1) |
|  | *Rattus norvegicus* | Fas death domain-associated protein | | | [NP_543167](http://www.ncbi.nlm.nih.gov/entrez/viewer.fcgi?val=NP_543167.1) |
| *BmTRAF3* | *Homo sapiens* | TNF receptor-associated factor 3，transcript variant 2 | | | 145726 |
|  | *Homo sapiens* | TNF receptor-associated factor 3，transcript variant 3 | | | 003300 |
|  | *Homo sapiens* | TNF receptor-associated factor 3, transcript variant 1, | | | 145725 |
|  | *Pan troglodytes* | TNF receptor-associated factor 3, transcript variant 2 | | | 001164779 |
|  | *Mus musculus* | TNF receptor-associated factor 3, transcript variant 2 | | | 001048206 |
|  | *Mus musculus* | TNF receptor-associated factor 3, transcript variant 1 | | | 011632 |
|  | *Danio rerio* | TNF receptor-associated factor 3 | | | 001003513 |
|  | *Rattus norvegicus* | Tnf receptor-associated factor 3 | | | 001108724 |
|  | *Bos taurus* | similar to Tnf receptor-associated factor 3 | | | 582595 |
| *BmTRAF6* | *Mus musculus* | TNF receptor-associated factor 6 | | | 009424 |
|  | *Homo sapiens* | TNF receptor-associated factor 6, transcript variant 2 | | | 004620 |
|  | *Homo sapiens* | TNF receptor-associated factor 6, transcript variant 1 | | | 145803 |
|  | *Xenopus (Silurana)* | TNF receptor-associated factor 6 | | | 001008161 |
|  | *Sus scrofa* | TNF receptor-associated factor 6 | | | 001105286 |
|  | *Rattus norvegicus* | Tnf receptor-associated factor 6 | | | 001107754 |
|  | *Danio rerio* | TNF receptor-associated factor 6 | | | 001044752 |
| ***Bcl-2 Family*** | | | | | |
| *BmBuffy* | *Homo sapiens* | B-cell lymphoma protein 2 alpha isoform | | | NP_000624 |
|  | *Homo sapiens* | B-cell lymphoma protein 2 beta isoform | | | NP_000648 |
|  | *Oryctolagus cuniculus* | B-cell CLL/lymphoma 2 protein | | | ABF71071 |
|  | *Mus musculus* | B-cell leukemia/lymphoma 2 isoform 1 | | | NP_033871 |
|  | *Mus musculus* | B-cell leukemia/lymphoma 2 isoform 2 | | | NP_803129 |
|  | *Rattus norvegicus* | B-cell leukemia/lymphoma 2 | | | NP_058689 |
|  | *Drosophila melanogaster* | Buffy CG8238-PA | | | NP_523702 |
|  | *Caenorhabditis elegans* | CEll Death abnormality family member (ced-9) | | | NP_499284 |
| ***Caspase Family*** | | | | | |
| *BmCaspase-1* | *Homo sapiens* | caspase 1 isoform α precursor | | | NP_150634 |
|  | *Homo sapiens* | caspase 1 isoform beta precursor | | | NP_001214 |
|  | *Homo sapiens* | caspase 1 isoform gamma precursor | | | NP_150635 |
|  | *Homo sapiens* | caspase 1 isoform epsilon | | | NP_150637 |
|  | *Homo sapiens* | caspase 1 isoform δ | | | NP_150636 |
|  | ***Bombyx mori*** | **Caspase 1** | | | **NP_001037050** |
|  | *Rattus norvegicus* | Caspase 1 | | | NP_036894 |
|  | *Drosophila melanogaster* | Death caspase-1 CG5370-PA | | | NP_476974 |
|  | *Gallus gallus* | caspase 1 | | | NP_990255 |
|  | *Sus scrofa* | caspase 1 | | | NP_999327 |
| *BmIce* | *Homo sapiens* | caspase 3 | | | NP_004337 |
|  | *Gallus gallus* | caspase 3 | | | NP_990056 |
|  | *Mus musculus* | caspase 3 | | | NP_033940 |
|  | *Rattus norvegicus* | caspase 3 | | | NP_037054 |
|  | *Sus scrofa* | caspase 3 | | | NP_999296 |
|  | *Canis lupus familiaris* | caspase 3 | | | NP_001003042 |
|  | *Drosophila melanogaster* | death executioner caspase related to Apopain/Yama CG14902-PA | | | NP_477462 |
|  | *Pan troglodytes* | caspase-3 | | | NP_001012435 |
|  | *Ovis aries* | caspase-3 | | | AAC25713 |
|  | ***Bombyx mori*** | **ICE** | | | **NP_001037297** |
|  | ***Bombyx mori*** | **ICE-2** | | | **ABC94941** |
|  | ***Bombyx mori*** | **ICE-5** | | | **ABC94942.1** |
| *BmDredd* | *Drosophila melanogaster* | death related ced-3/Nedd2-like protein, isoform D | | | NP_477251 |
|  | ***Bombyx mori*** | **death related ced-3/Nedd2-like protein** | | | **NP_001108337** |
|  | *Homo sapiens* | caspase 8 isoform A precursor | | | NP_001219 |
|  | *Danio rerio* | caspase 8, apoptosis-related cysteine peptidase | | | NP_571585 |
|  | *Rattus norvegicus* | caspase 8 | | | NP_071613 |
|  | *Gallus gallus* | caspase 8 | | | NP_989923 |
|  | *Mus musculus* | caspase 8 | | | NP_001073595 |
|  | *Canis lupus familiaris* | caspase 8 | | | NP_001041494 |
|  | *Sus scrofa* | caspase-8 | | | NP_001026949 |
|  | *Pan troglodytes* | PREDICTED: caspase 8 isoform 1 | | | XP_001172044 |
| *BmDronc* | *Rattus norvegicus* | caspase 9 | | | NP_113820 |
|  | *Homo sapiens* | caspase 9 isoform alpha preproprotein | | | NP_001220 |
|  | *Homo sapiens* | caspase 9 isoform beta preproprotein | | | NP_127463 |
|  | *Canis familiaris* | PREDICTED: caspase 9, apoptosis-related cysteine protease isoform 2 | | | XP_852163 |
|  | *Canis familiaris* | PREDICTED: caspase 9, apoptosis-related cysteine protease isoform 3 | | | XP_865164 |
|  | *Mus musculus* | caspase 9 | | | NP_056548 |
|  | *Xenopus laevis* | caspase-9 | | | NP_001079035 |
|  | *Gallus gallus* | PREDICTED: hypothetical protein | | | XP_424580 |
|  | *Pan troglodytes* | PREDICTED: caspase 9 isoform 1 | | | XP_001149139 |
|  | *Pan troglodytes* | PREDICTED: caspase 9 isoform 2 | | | XP_001149405 |
|  | *Pan troglodytes* | PREDICTED: caspase 9 isoform 3 | | | XP_001149481 |
|  | *Pan troglodytes* | PREDICTED: similar to Caspase 9, apoptosis-related cysteine peptidase isoform 4 | | | XP_001149560 |
|  | *Pan troglodytes* | PREDICTED: caspase 9 isoform 5 | | | XP_513049 |
|  | *Felis catus* | CASP9 | | | ABJ16421 |
|  | *Drosophila melanogaster* | Nedd2-like caspase CG8091-PA | | | NP_524017 |
|  | *Aedes aegypti* | ecdysone-inducible caspase, putative | | | XP_001655433 |
| ***IAP Faimily*** | | | | | |
| *BmIAP* | *Neodiprion sertifer nucleopolyhedrovirus* | | | inhibitor of apoptosis; iap | YP_025124 |
|  | ***Bombyx mori*** | | | **inhibitor of apoptosis protein** | **NP_001037024** |
|  | *Drosophila melanogaster* | | | thread, isoform B | NP_730097 |
|  | *Drosophila melanogaster* | | | thread, isoform A | NP_524101 |
|  | *Drosophila melanogaster* | | | thread, isoform C | NP_730098 |
| *BmIAP2* | *Hyphantria cunea nucleopolyhedrovirus* | | | IAP2 | YP_473268 |
|  | *Bombyx mori NPV* | | | IAP2 | NP_047474 |
|  | *Drosophila melanogaster* | | | inhibitor of apoptosis 2, isoform B | NP_788362 |
|  | *Drosophila melanogaster* | | | inhibitor of apoptosis 2, isoform A | NP_477127 |
|  | *Orgyia leucostigma NPV* | | | inhibitor of apoptosis protein 2 | YP_001650953 |
| *BmSurvivin-1* | *Danio rerio* | | | baculoviral IAP repeat-containing 5A | NP_919378 |
|  | *Xenopus (Silurana) tropicalis* | | | baculoviral IAP repeat-containing 5 | NP_001037948 |
|  | *Mus musculus* | | | baculoviral IAP repeat-containing 5 isoform 3 | NP_001012273 |
|  | *Mus musculus* | | | baculoviral IAP repeat-containing 5 isoform 1 | NP_033819 |
|  | *Homo sapiens* | | | baculoviral IAP repeat-containing protein 5 isoform 2 | NP_001012270 |
|  | *Homo sapiens* | | | baculoviral IAP repeat-containing protein 5 isoform 3 | NP_001012271 |
|  | *Homo sapiens* | | | baculoviral IAP repeat-containing protein 5 isoform 1 | NP_001159 |
|  | *Macaca mulatta* | | | PREDICTED: baculoviral IAP repeat-containing 5 | XP_001083183 |
| *BmSurvivin-2* | *Danio rerio* | | | baculoviral IAP repeat-containing 5B | NP_660196 |
|  | *Schizosaccharomyces japonicus yFS275* | | | survivin 2 | XP_002175521 |
| ***Other Regulators and Executors*** | | | | | |
| *BmAcinus* | *Danio rerio* | | PREDICTED: apoptotic chromatin condensation inducer 1b | | XP_694912 |
|  | *Danio rerio* | | apoptotic chromatin condensation inducer 1a | | NP_001007106 |
|  | *Mus musculus* | | apoptotic chromatin condensation inducer 1 isoform 2 | | NP_075679 |
|  | *Mus musculus* | | apoptotic chromatin condensation inducer 1 isoform 3 | | NP_001078941 |
|  | *Mus musculus* | | apoptotic chromatin condensation inducer 1 isoform 4 | | NP_001078942 |
|  | *Mus musculus* | | apoptotic chromatin condensation inducer 1 isoform 1 | | NP_062513 |
|  | *Homo sapiens* | | apoptotic chromatin condensation inducer 1 | | NP_055792 |
| *BmAIF* | *Danio rerio* | | programmed cell death 8 | | NP_956396 |
|  | *Rattus norvegicus* | | programmed cell death 8 | | NP_112646 |
|  | *Caenorhabditis elegans* | | Worm AIF (apoptosis inducing factor) Homolog family member (wah-1) | | NP_499564 |
|  | *Dictyostelium discoideum AX4* | | hypothetical protein DDB_0191137 | | XP_636815 |
|  | *Drosophila melanogaster* | | CG7263-PB, isoform B | | NP_722765 |
|  | *Drosophila melanogaster* | | CG7263-PA, isoform A | | NP_608649 |
| *BmAkt* | *Homo sapiens* | | v-akt murine thymoma viral oncogene homolog 1 | | NP_001014431 |
|  | *Homo sapiens* | | v-akt murine thymoma viral oncogene homolog 1 | | NP_001014432 |
|  | *Homo sapiens* | | v-akt murine thymoma viral oncogene homolog 1 | | NP_005154 |
|  | *Rattus norvegicus* | | thymoma viral proto-oncogene 1 | | NP_150233 |
|  | *Caenorhabditis elegans* | | AKT kinase family member (akt-1) | | NP_001023646 |
|  | *Caenorhabditis elegans* | | AKT kinase family member (akt-1) | | NP_001023645 |
|  | *Caenorhabditis elegans* | | AKT kinase family member (akt-1) | | NP_001023647 |
|  | *Mus musculus* | | thymoma viral proto-oncogene 1 | | NP_033782 |
|  | *Mus musculus* | | thymoma viral proto-oncogene 2 | | NP_031460 |
|  | *Drosophila melanogaster* | | Akt1 CG4006-PA, isoform A | | NP_732114 |
|  | *Drosophila melanogaster* | | Akt1 CG4006-PA, isoform B | | NP_732115 |
|  | *Drosophila melanogaster* | | Akt1 CG4006-PC, isoform C | | NP_732113 |
| *BmApaf-1* | *Homo sapiens* | | apoptotic peptidase activating factor 1 isoform b | | NP_001151 |
|  | *Homo sapiens* | | apoptotic peptidase activating factor 1 isoform a | | NP_037361 |
|  | *Mus musculus* | | apoptotic protease activating factor 1 | | NP_001036023 |
|  | *Mus musculus* | | apoptotic protease activating factor 1 | | NP_033814 |
|  | *Rattus norvegicus* | | apoptotic protease activating factor 1 | | NP_076469 |
|  | *Caenorhabditis elegans* | | CEll Death abnormality family member (ced-4) | | NP_001021202 |
|  | *Caenorhabditis elegans* | | CEll Death abnormality family member (ced-4) | | NP_001021203 |
|  | *Drosophila melanogaster* | | Apaf-1-related-killer CG6829-PB, isoform B | | NP_725637 |
|  | *Drosophila melanogaster* | | Apaf-1-related-killer CG6829-PB, isoform A | | NP_725638 |
| *BmApp* | *Mus musculus* | | amyloid beta (A4) precursor protein | | NP_031497 |
|  | *Homo sapiens* | | amyloid beta A4 protein precursor, isoform c | | NP_958817 |
|  | *Homo sapiens* | | amyloid beta A4 protein precursor, isoform b | | NP_958816 |
|  | *Homo sapiens* | | amyloid beta A4 protein precursor, isoform a | | NP_000475 |
|  | *Bos taurus* | | amyloid beta (A4) precursor protein | | NP_001070264 |
|  | *Gallus gallus* | | amyloid beta A4 protein | | NP_989639 |
|  | *Sus scrofa* | | amyloid beta A4 protein | | NP_999537 |
|  | *Rattus norvegicus* | | amyloid beta (A4) precursor protein | | NP_062161 |
|  | *Pan troglodytes* | | amyloid beta A4 protein | | NP_001013036 |
|  | *Arabidopsis thaliana* | | APP (ARABIDOPSIS POLY(ADP-RIBOSE) POLYMERASE) | | NP_192148 |
|  | *Arabidopsis thaliana* | | ASK1 | | NP_172563 |
| *BmAsk1* | *Pichia stipitis CBS 6054* | | DASH complex subunit ask1 | | XP_001387495 |
|  | *Saccharomyces cerevisiae* | | Ask1p | | NP_012872 |
|  | *Homo sapiens* | | mitogen-activated protein kinase kinase kinase 5 | | NP_005914 |
|  | *Mus musculus* | | mitogen activated protein kinase kinase kinase 5 | | NP_032606 |
|  | *Drosophila melanogaster* | | Protein kinase at 92B CG4720-PB, isoform B | | NP_996240 |
|  | *Drosophila melanogaster* | | Protein kinase at 92B CG4720-PA, isoform A | | NP_477089 |
|  | *Danio rerio* | | PREDICTED: mitogen-activated protein kinase kinase kinase 5 | | XP_683387 |
| *BmAtf2* | *Homo sapiens* | | activating transcription factor 2 | | NP_001871 |
|  | *Mus musculus* | | activating transcription factor 2 isoform 1 | | NP_001020264 |
|  | *Mus musculus* | | activating transcription factor 2 isoform 2 | | NP_033845 |
|  | *Rattus norvegicus* | | activating transcription factor 2 | | NP_112280 |
|  | *Canis familiaris* | | similar to activating transcription factor 2 isoform 2 | | XP_861588 |
|  | *Canis familiaris* | | similar to activating transcription factor 2 isoform 1 | | XP_535970 |
|  | *Gallus gallus* | | activating transcription factor 2 | | NP_990235 |
|  | *Xenopus laevis* | | activating transcription factor 2 | | NP_001079255 |
| *BmCdc2* | *Homo sapiens* | | cell division cycle 2 protein isoform 1 | | NP_001777 |
|  | *Homo sapiens* | | cell division cycle 2 protein isoform 2 | | NP_203698 |
|  | ***Bombyx mori*** | | **Bm cdc2** | | **NP_001037512** |
|  | *Drosophila melanogaster* | | cdc2 CG5363-PA | | NP_476797 |
|  | *Macaca mulatta* | | PREDICTED: cell division cycle 2, G1 to S and G2 to M isoform 1 | | XP_001095697 |
|  | *Macaca mulatta* | | PREDICTED: cell division cycle 2 protein isoform 2 | | XP_001095798 |
|  | *Macaca mulatta* | | PREDICTED: cell division cycle 2 protein isoform 3 | | XP_001095903 |
|  | *Danio rerio* | | cell division cycle 2 | | NP_997729 |
|  | *Xenopus laevis* | | Cell division control protein 2 homolog 1 | | NP_001080554 |
|  | *Xenopus tropicalis* | | cell division cycle 2 | | NP_988908 |
| *BmCreb* | *Homo sapiens* | | cAMP responsive element binding protein 1 isoform B | | NP_604391 |
|  | *Caenorhabditis elegans* | | CREB Homolog family member (crh-1) | | NP_001022862 |
| *BmCyt C* | *Drosophila melanogaster* | | cytochrome c proximal | | NP_477176 |
|  | *Drosophila melanogaster* | | cytochrome c distal | | NP_477164 |
|  | *Rattus norvegicus* | | cytochrome c, somatic | | NP_036971 |
|  | *Homo sapiens* | | cytochrome c | | NP_061820 |
|  | *Bordetella petrii DSM 12804* | | cytochrome c family protein | | YP_001629280 |
|  | *Mus musculus* | | cytochrome c, somatic | | NP_031834 |
|  | *Xenopus (Silurana) tropicalis* | | cytochrome c, somatic | | NP_988895 |
|  | *Gallus gallus* | | cytochrome c, somatic | | NP_001072946 |
|  | *Pan troglodytes* | | cytochrome c, somatic | | NP_001065289 |
|  | *Taeniopygia guttata* | | cytochrome c, somatic | | NP_001137145 |
|  | *Sus scrofa* | | cytochrome c, somatic | | NP_001123442 |
|  | *Xenopus laevis* | | cytochrome c, somatic | | NP_001086101 |
|  | *Pongo abelii* | | cytochrome c, somatic | | NP_001124639 |
|  | *Bos taurus* | | cytochrome c, somatic | | NP_001039526 |
|  | ***Bombyx mori*** | | **mitochondrial cytochrome c** | | **ACF41193** |
| *BmDapk* | *Homo sapiens* | | death-associated protein kinase 1 | | NP_004929 |
|  | *Mus musculus* | | death associated protein kinase 1 | | NP_083929 |
|  | *Rattus norvegicus* | | Death-associated protein kinase 3 | | NP_001100805 |
|  | *Caenorhabditis elegans* | | DAP (Death-Associated Protein) Kinase homolog family member | | NP_490840 |
| *BmEndo G* | *Mus musculus* | | endonuclease G | | NP_031957 |
|  | *Rattus norvegicus* | | endonuclease G | | NP_001030110 |
|  | *Homo sapiens* | | endonuclease G precursor | | NP_004426 |
|  | *Bos taurus* | | endonuclease G | | NP_787017 |
|  | *Xenopus tropicalis* | | endonuclease G | | NP_001017202 |
|  | *Ovis aries* | | endonuclease G | | AAP46140 |
|  | *Pan troglodytes* | | PREDICTED: similar to Endonuclease G | | ABJ16427 |
|  | *Gallus gallus* | | PREDICTED: similar to Endonuclease G | | XP_415487 |
|  | *Felis catus* | | ENDOG | | ABJ16423 |
|  | *Rattus norvegicus* | | mitogen-activated protein kinase 3 | | NP_059043 |
| *BmErk* | *Pichia stipitis CBS 6054* | | Extracellular signal-regulated kinase 1 | | XP_001387701 |
|  | *Homo sapiens* | | mitogen-activated protein kinase 3 | | NP_001035145 |
|  | *Homo sapiens* | | mitogen-activated protein kinase 3 | | NP_002737 |
|  | *Mus musculus* | | mitogen-activated protein kinase 3 | | NP_036082 |
|  | *Danio rerio* | | mitogen-activated protein kinase 3 | | NP_958915 |
|  | ***Bombyx mori*** | | **Extracellular regulated MAP kinase** | | **NP_001036921** |
| *BmFkhr* | *Drosophila melanogaster* | | forkhead box, sub-group O CG3143-PA, isoform A | | NP_650330 |
|  | *Drosophila melanogaster* | | forkhead box, sub-group O CG3143-PC, isoform C | | NP_996204 |
|  | *Drosophila melanogaster* | | forkhead box, sub-group O CG3143-PB, isoform B | | NP_996205 |
|  | *Danio rerio* | | forkhead box O5 | | NP_571160 |
|  | *Mus musculus* | | forkhead box O1 | | NP_062713 |
|  | *Rattus norvegicus* | | forkhead box G1 | | NP_036692 |
|  | *Homo sapiens* | | forkhead box O1 | | NP_002006 |
| *BmGas2* | *Homo sapiens* | | growth arrest-specific 2 | | NP_005247 |
|  | *Mus musculus* | | growth arrest specific 2 | | NP_032113 |
|  | *Pan troglodytes* | | PREDICTED: growth arrest-specific 2 isoform 1 | | XP_001173903 |
|  | *Pan troglodytes* | | PREDICTED: growth arrest-specific 2 isoform 2 | | XP_001173919 |
|  | *Pan troglodytes* | | PREDICTED: growth arrest-specific 2 isoform 3 | | XP_001173929 |
|  | *Pan troglodytes* | | PREDICTED: growth arrest-specific 2 isoform 4 | | XP_001173934 |
|  | *Pan troglodytes* | | PREDICTED: growth arrest-specific 2 isoform 5 | | XP_001173940 |
|  | *Pan troglodytes* | | PREDICTED: growth arrest-specific 2 isoform 6 | | XP_508333 |
|  | *Canis familiaris* | | PREDICTED: similar to growth arrest-specific 2 isoform 2 | | XP_865617 |
|  | *Canis familiaris* | | PREDICTED: similar to growth arrest-specific 2 isoform 1 | | XP_534091 |
| *BmGsk3* | *Caenorhabditis elegans* | | Glycogen Synthase Kinase family member (gsk-3) | | NP_493243 |
|  | *Drosophila melanogaster* | | shaggy CG2621-PL, isoform L | | NP_001036259 |
|  | *Drosophila melanogaster* | | shaggy CG2621-PD, isoform D | | NP_476716 |
|  | *Drosophila melanogaster* | | shaggy CG2621-PA, isoform A | | NP_476714 |
|  | *Drosophila melanogaster* | | shaggy CG2621-PJ, isoform J | | NP_996336 |
|  | *Drosophila melanogaster* | | shaggy CG2621-PF, isoform F | | NP_726823 |
|  | *Drosophila melanogaster* | | shaggy CG2621-PE, isoform E | | NP_726822 |
|  | *Drosophila melanogaster* | | shaggy CG2621-PB, isoform B | | NP_476715 |
|  | *Drosophila melanogaster* | | shaggy CG2621-PI, isoform I | | NP_996337 |
|  | *Drosophila melanogaster* | | shaggy CG2621-PH, isoform H | | NP_996338 |
|  | *Drosophila melanogaster* | | shaggy CG2621-PC, isoform C | | NP_599105 |
|  | *Drosophila melanogaster* | | shaggy CG2621-PG, isoform G | | NP_996335 |
|  | *Drosophila melanogaster* | | shaggy CG2621-PK, isoform K | | NP_996334 |
|  |  |  |  |  |  |
| *BmHtra2* | *Drosophila melanogaster* | | HtrA2 | | NP_650366 |
|  | *Leptospira interrogans serovar Lai str. 56601* | | HtrA2 | | NP_713130 |
|  | *Leptospira interrogans serovar Copenhageni str. Fiocruz L1-130* | | HtrA2 | | YP_001081 |
|  | *Homo sapiens* | | HtrA serine peptidase 2 isoform 1 preproprotein | | NP_037379 |
|  | *Homo sapiens* | | HtrA serine peptidase 2 isoform 2 | | NP_659540 |
| *BmIcad* | ***Bombyx mori*** | | **cell death activator CIDE-B** | | **NP_001108342** |
| *BmJnk* | ***Bombyx mori*** | | **c-Jun NH2-terminal kinase** | | **NP_001103396** |
| *BmMkk7* | *Homo sapiens* | | mitogen-activated protein kinase kinase 7 | | NP_660186 |
|  | *Xenopus laevis* | | mitogen-activated protein kinase kinase 7 | | NP_001081118 |
|  | *Drosophila melanogaster* | | hemipterous CG4353-PA, isoform A | | NP_727661 |
|  | *Arabidopsis thaliana* | | ATMKK7 (MAP KINASE KINASE7) | | NP_173271 |
| *BmP53* | *Homo sapiens* | | tumor protein p53 isoform a | | NP_000537 |
|  | *Homo sapiens* | | tumor protein p53 isoform a | | NP_001119584 |
|  | *Rattus norvegicus* | | transformation related protein 53 | | NP_112251 |
|  | *Drosophila melanogaster* | | p53, isoform B | | NP_996267 |
|  | *Drosophila melanogaster* | | p53, isoform A | | NP_996268 |
|  | *Danio rerio* | | tumor protein p53 | | NP_571402 |
|  | *Ovis aries* | | P53 protein | | NP_001009403 |
| *BmP70s6k* | *Homo sapiens* | | ribosomal protein S6 kinase, 70kDa, polypeptide 2 | | NP_003943 |
|  | *Homo sapiens* | | ribosomal protein S6 kinase, 70kDa, polypeptide 1 | | NP_003152 |
|  | *Oryctolagus cuniculus* | | ribosomal protein S6 kinase, 70kDa, polypeptide 1 | | NP_001095160 |
|  | *Rattus norvegicus* | | ribosomal protein S6 kinase, polypeptide 2 | | NP_001010962 |
|  | *Mus musculus* | | ribosomal protein S6 kinase, polypeptide 2 | | NP_067460 |
|  | *Mus musculus* | | ribosomal protein S6 kinase, polypeptide 1 | | NP_082535 |
|  | *Drosophila melanogaster* | | RPS6-p70-protein kinase CG10539-PA | | NP_523941 |
|  | *Bos taurus* | | ribosomal protein S6 kinase, 70kDa, polypeptide 1 | | NP_991385 |
| *BmP90srk* | *Homo sapiens* | | ribosomal protein S6 kinase, 90kDa, polypeptide 2 isoform a | | NP_066958 |
|  | *Homo sapiens* | | ribosomal protein S6 kinase, 90kDa, polypeptide 2 isoform b | | NP_001006933 |
|  | *Rattus norvegicus* | | PREDICTED: similar to Ribosomal protein S6 kinase alpha 2 | | XP_001058582.1 |
|  | *Rattus norvegicus* | | PREDICTED: similar to Ribosomal protein S6 kinase alpha 2 | | XP_341759 |
|  | *Mus musculus* | | ribosomal protein S6 kinase polypeptide 1 | | NP_033123 |
|  | *Mus musculus* | | ribosomal protein S6 kinase polypeptide 2 | | NP_035429 |
| *BmPax6* | *Mus musculus* | | paired box gene 6 | | NP_038655 |
|  | *Gallus gallus* | | paired box gene 6 | | NP_990397 |
|  | *Ciona intestinalis* | | Pax6 protein | | NP_001027641 |
|  | *Oryctolagus cuniculus* | | paired box protein PAX6 isoform b | | NP_001075686 |
|  | *Homo sapiens* | | paired box gene 6 isoform a | | NP_000271 |
|  | *Homo sapiens* | | paired box gene 6 isoform b | | NP_001595 |
|  | *Bos taurus* | | paired box gene 6 | | NP_001035735 |
|  | *Danio rerio* | | paired box gene 6a | | NP_571379 |
|  | *Drosophila melanogaster* | | eyeless CG1464-PB, isoform B | | NP_726607 |
|  | *Drosophila melanogaster* | | eyeless CG1464-PD, isoform D | | NP_001014693 |
|  | *Drosophila melanogaster* | | eyeless CG1464-PA, isoform A | | NP_524628 |
|  | *Drosophila melanogaster* | | eyeless CG1464-PC, isoform C | | NP_001014694 |
|  | *Canis lupus familiaris* | | paired box gene 6 | | NP_001091013 |
|  | *Rattus norvegicus* | | paired box gene 6 | | NP_037133 |
| *BmParp* | *Drosophila melanogaster* | | CG40411-PD.3 | | NP_001015397 |
|  | *Drosophila melanogaster* | | CG40411-PE.3 | | NP_001015395 |
|  | *Drosophila melanogaster* | | CG40411-PC.3 | | NP_001015396 |
|  | *Homo sapiens* | | poly (ADP-ribose) polymerase family, member 1 | | NP_001609 |
|  | *Mus musculus* | | poly (ADP-ribose) polymerase family, member 1 | | NP_031441 |
| *BmPdk* | *Arabidopsis thaliana* | | PDK (PYRUVATE DEHYDROGENASE KINASE) | | NP_187300 |
|  | ***Bombyx mori*** | | **pyruvate dehydrogenase kinase** | | **NP_001108115** |
|  | *Homo sapiens* | | pyruvate dehydrogenase kinase, isozyme 1 | |  |
|  | *Mus musculus* | | pyruvate dehydrogenase kinase, isoenzyme 1 | |  |
|  | *Rattus norvegicus* | | pyruvate dehydrogenase kinase, isoenzyme 1 | |  |
|  | *Caenorhabditis elegans* | | PDK-class.1 protein kinase family member (pdk-1) | |  |
|  | *Caenorhabditis elegans* | | PDK-class.2 protein kinase family member (pdk-1) | |  |
| *BmPi3k* | *Mus musculus* | | phosphatidylinositol 3-kinase, regulatory subunit, polypeptide 1 isoform 1 | | NP_001070963 |
|  | *Mus musculus* | | phosphatidylinositol 3-kinase, regulatory subunit, polypeptide 1 isoform 2 | | NP_001020126 |
|  | *Homo sapiens* | | phosphoinositide-3-kinase, catalytic, alpha polypeptide | | NP_006209 |
|  | *Drosophila melanogaster* | | Phosphotidylinositol 3 kinase 68D CG11621-PC, isoform C | | NP_729743 |
|  | *Drosophila melanogaster* | | Phosphotidylinositol 3 kinase 68D CG11621-PA, isoform A | | NP_524028 |
|  | *Drosophila melanogaster* | | Phosphotidylinositol 3 kinase 68D CG11621-PB, isoform B | | NP_729745 |
| *BmPka* | *Mus musculus* | | protein kinase, cAMP dependent, catalytic, alpha | | NP_032880 |
|  | *Drosophila melanogaster* | | cAMP-dependent protein kinase R1 CG3263-PA, isoform A | | NP_730573 |
|  | ***Bombyx mori*** | | **cAMP-dependent protein kinase R2** | | **NP_001104823** |
| *BmPkc* | *Apis mellifera* | | PREDICTED: similar to Protein C kinase 53E CG6622-PB, isoform B isoform 1 | | XP_391874 |
|  | *Caenorhabditis elegans* | | Protein Kinase C family member (pkc-2) | | NP_001024518 |
|  | *Caenorhabditis elegans* | | Protein Kinase C family member (pkc-2) | | NP_001024516 |
|  | *Caenorhabditis elegans* | | Protein Kinase C family member (pkc-2) | | NP_001024517 |
|  | *Caenorhabditis elegans* | | Protein Kinase C family member (pkc-1) | | NP_506014 |
|  | *Caenorhabditis elegans* | | Protein Kinase C family member (pkc-3) | | XP_391874 |
|  | *Rattus norvegicus* | | protein kinase C, gamma | | NP_036760 |
|  | *Apis mellifera* | | PREDICTED: similar to Protein C kinase 53E CG6622-PB, isoform B isoform 1 | | XP_391874 |
|  | ***Bombyx mori*** | | **conventional protein kinase C** | | **NP_001036978** |
|  | *Homo sapiens* | | v-raf-1 murine leukemia viral oncogene homolog 1 | | NP_002871 |
| *BmRaf* | *Mus musculus* | | protein kinase raf 1 | | NP_084056 |
|  | *Mus musculus* | | v-raf murine sarcoma 3611 viral oncogene homolog | | NP_033833 |
|  | *Rattus norvegicus* | | v-raf murine sarcoma 3611 viral oncogene homolog 1 isoform 1 | | NP_071977 |
|  | *Rattus norvegicus* | | v-raf murine sarcoma 3611 viral oncogene homolog 1 isoform 2 | | NP_001028835 |
|  | *Xenopus laevis* | | raf protein | | NP_001081476 |
| *BmRas* | *Xenopus laevis* | | Ras protein | | NP_001081762 |
|  | *Drosophila melanogaster* | | raspberry, isoform C | | NP_727442 |
|  | *Drosophila melanogaster* | | raspberry, isoform A | | NP_727441 |
|  | *Drosophila melanogaster* | | raspberry, isoform B | | NP_524646 |
|  | ***Bombyx mori*** | | **ras oncogene** | | **NP_001036993** |
|  | ***Bombyx mori*** | | **ras oncogene** | | **NP_001036973** |
| *BmReaper* |  | |  | |  |
| *BmRock1* | *Homo sapiens* | | Rho-associated, coiled-coil containing protein kinase 1 | | NP_005397 |
|  | *Mus musculus* | | Rho-associated coiled-coil forming kinase 1 | | NP_033097 |
|  | *Macaca mulatta* | | PREDICTED: Rho-associated, coiled-coil containing protein kinase 1 isoform 2 | | XP_001091134 |
|  | *Macaca mulatta* | | PREDICTED: Rho-associated, coiled-coil containing protein kinase 1 isoform 3 | | XP_001091266 |
|  | *Macaca mulatta* | | PREDICTED: Rho-associated, coiled-coil containing protein kinase 1 isoform 1 | | XP_001090896 |
|  | *Gallus gallus* | | PREDICTED: similar to corneal epithelial Rho-associated-ser/thr kinase; ROCK-I | | XP_419151 |
|  | *Sus scrofa* | | Rho-kinase beta | | BAA20905 |
|  | *Canis familiaris* | | PREDICTED: similar to Rho-associated protein kinase 1 (Rho-associated, coiled-coil containing protein kinase 1) (p160 ROCK-1) (p160ROCK) | | XP_537305 |
|  | *Oryctolagus cuniculus* | | Rho-associated, coiled-coil containing protein kinase 1 | | NP_001075836 |
| *BmSir2* | *Drosophila melanogaster* | | Sir2 CG5216-PA | | NP_477351 |
|  | *Xenopus laevis* | | sirtuin 2 | | NP_001088636 |
|  | *Pichia stipitis CBS 6054* | | NAD-dependent histone deacetylase | | XP_001387128 |
|  | *Methanopyrus kandleri AV19* | | NAD-dependent protein deacetylase, SIR2 family | | NP_614358 |
|  | *Haloarcula marismortui*  *ATCC 43049* | | transcriptional regulator Sir2 family | | YP_135203 |
|  | *Lactobacillus salivarius*  *subsp. salivarius UCC118* | | SIR2 family protein | | YP_535101 |
|  | *Thermoanaerobacter tengcongensis MB4* | | NAD-dependent protein deacetylase, Sir2 family | | NP_623170 |
|  | *Pelotomaculum thermopropionicum SI* | | NAD-dependent protein deacetylases | | YP_001212569 |
|  | ***Bombyx mori*** | | **sid-1-related gene3** | | **NP_001106736** |
|  | ***Bombyx mori*** | | **sid-1-related gene1** | | **NP_001106735** |
|  | ***Bombyx mori*** | | **sid-1-related gene2** | | **100134918** |
| *BmStat* | *Canis familiaris* | | PREDICTED: similar to signal transducer and activator of transcription 1 isoform beta isoform 4 | | XP_855956 |
|  | *Canis familiaris* | | PREDICTED: similar to Signal transducer and activator of transcription 1-alpha/beta isoform 5 | | XP_856037 |
|  | *Canis familiaris* | | PREDICTED: similar to Signal transducer and activator of transcription 1-alpha/beta isoform 1 | | XP_545571 |
|  | *Canis familiaris* | | PREDICTED: similar to signal transducer and activator of transcription 1 isoform beta isoform 3 | | XP_855870 |
|  | *Canis familiaris* | | PREDICTED: similar to Signal transducer and activator of transcription 1-alpha/beta isoform 2 | | XP_848353 |
|  | *Rattus norvegicus* | | signal transducer and activator of transcription 1 isoform alpha | | NP_116001 |
|  | *Rattus norvegicus* | | signal transducer and activator of transcription 1 isoform beta | | NP_001029336 |
|  | *Bos taurus* | | signal transducer and activator of transcription 1 | | NP_001071368 |
|  | *Homo sapiens* | | signal transducer and activator of transcription 1 isoform beta | | NP_644671 |
|  | *Homo sapiens* | | signal transducer and activator of transcription 1 isoform alpha | | NP_009330 |
|  | *Sus scrofa* | | signal transducer and activator of transcription 1 | | NP_998934 |
|  | *Danio rerio* | | signal transducer and activator of transcription 1 | | NP_571555 |
|  | *Xenopus tropicalis* | | signal transducer and activator of transcription 1 | | NP_001072935 |
|  | *Mus musculus* | | signal transducer and activator of transcription 1 | | NP_033309 |
|  | ***Bombyx mori*** | | **signal transducer and activator of transcription** | | **ACR61178** |
| *BmTak1* | *Homo sapiens* | | mitogen-activated protein kinase kinase kinase 7 interacting protein 1 isoform alpha | | NP_006107 |
|  | *Homo sapiens* | | mitogen-activated protein kinase kinase kinase 7 interacting protein 1 isoform beta | | NP_705717 |
|  | *Mus musculus* | | mitogen-activated protein kinase kinase kinase 7 interacting protein 1 | | NP_079885 |
|  | *Xenopus tropicalis* | | mitogen-activated protein kinase kinase kinase 7 interacting protein 1 | | NP_001016463 |
