# Supplementary figures and images for: The genomic underpinnings of apoptosis in the silkworm, Bombyx mori
Source: BMC Genomics. 2010 Oct 31;11:611. doi: 10.1186/1471-2164-11-611 (PMC3091752; doi:10.1186/1471-2164-11-611)

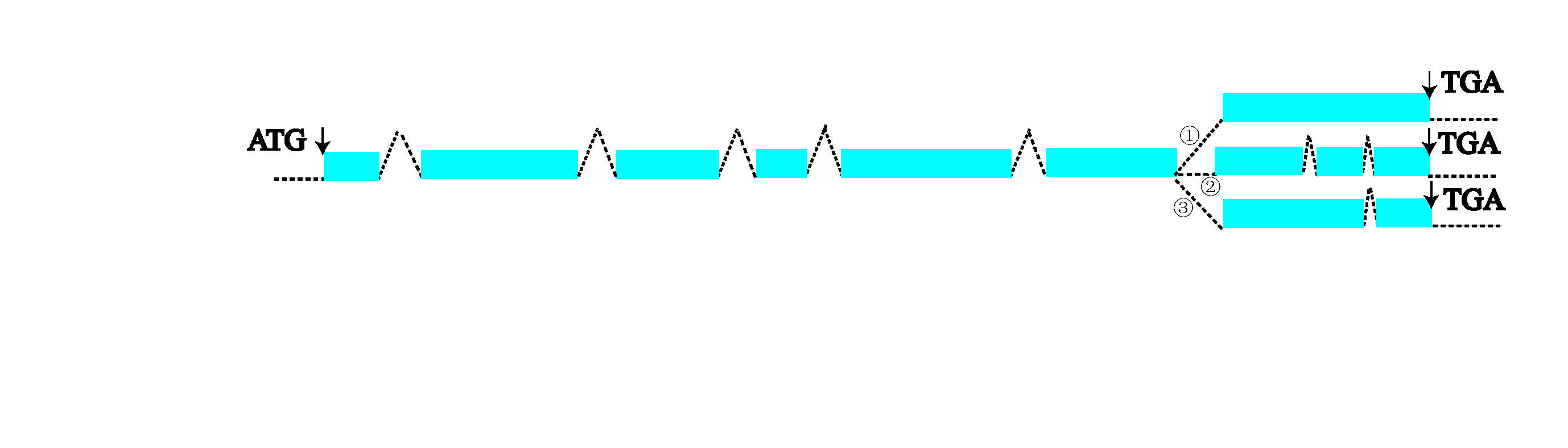

Supplement: Additional file 4 — Three different splice variants of BmICE in Bombyx mori. 1, 2 and 3 are the splice variants of BmICE, named BmICE, BmICE-5 and BmICE-2 respectively. [file 1471-2164-11-611-S4.JPEG]
